# Cancer-associated fibroblasts as therapeutic targets for cancer: advances, challenges, and future prospects

**DOI:** 10.1186/s12929-024-01099-2

**Published:** 2025-01-09

**Authors:** Zhipeng Cao, Sadia Quazi, Sakshi Arora, Laura D. Osellame, Ingrid J. Burvenich, Peter W. Janes, Andrew M. Scott

**Affiliations:** 1 Tumour Targeting Laboratory, Olivia Newton-John Cancer Research Institute, Melbourne, VIC 3084 Australia; 2 School of Cancer Medicine, La Trobe University, Melbourne, VIC 3086 Australia; 3 Department of Molecular Imaging and Therapy, Austin Health, Melbourne, VIC 3084 Australia; 4 Biomedicine Discovery Institute and Department of Biochemistry and Molecular Biology, Monash University, Melbourne, VIC 3800 Australia; 5 Department of Medicine, University of Melbourne, Melbourne, VIC 3010 Australia

**Keywords:** Tumour microenvironment, Cancer-associated fibroblasts, Tumour stroma, Targeted therapy, Clinical trials

## Abstract

Research into cancer treatment has been mainly focused on developing therapies to directly target cancer cells. Over the past decade, extensive studies have revealed critical roles of the tumour microenvironment (TME) in cancer initiation, progression, and drug resistance. Notably, cancer-associated fibroblasts (CAFs) have emerged as one of the primary contributors in shaping TME, creating a favourable environment for cancer development. Many preclinical studies have identified promising targets on CAFs, demonstrating remarkable efficacy of some CAF-targeted treatments in preclinical models. Encouraged by these compelling findings, therapeutic strategies have now advanced into clinical evaluation. We aim to provide a comprehensive review of relevant subjects on CAFs, including CAF-related markers and targets, their multifaceted roles, and current landscape of ongoing clinical trials. This knowledge can guide future research on CAFs and advocate for clinical investigations targeting CAFs.

## Introduction

The tumour microenvironment (TME) has emerged as a pivotal player in cancer development and drug resistance. With the introduction of the "seed and soil" theory in 1989 by Stephen Paget [1], the significance of TME has grown considerably. Substantial evidence now indicates that diverse cell populations within tumours create a supportive environment for the survival, growth, and metastasis of cancer cells. In line with the "seed and soil" theory, tumour cells ("seed") preferentially grow in organs with a suitable microenvironment ("soil"), leading to a non-random distribution of metastasis among organs. It is now widely accepted that cancer behaviours are regulated by both intrinsic factors and the intricate TME. The TME constitutes a complex and dynamic niche formed by various cellular and molecular components engaging in communication and interactions with cancer cells. Through these inter-TME dialogues and crosstalk with cancer cells, the TME provide a nurturing and protective environment for cancer cells.

The TME involves two major cellular components: immune cells and stromal cells. TME immune cells include myeloid-derived suppressor cells (MDSC), tumour-associated macrophages (TAMs), tumour-associated neutrophils, regulatory T (Treg) cells, natural killer cells, dendritic cells, B cells, effector T cells and T helper cells. The roles of these immune cells in the TME and cancer development have been thoroughly reviewed elsewhere [2–6], and will not be discussed here. The other large proportion of cells within TME are stromal cells, and the tumour-stroma ratio has shown promise as a prognostic biomarker for certain cancer types in clinical settings [7–11]. TME stromal cells include endothelial cells (blood vessels), as well as inter-related cancer-associated fibroblasts (CAFs), mesenchymal stromal cells (MSCs), and pericytes, which all share certain characteristics and plasticity, being able to interconvert and differentiate into different cell types [12, 13]. The roles of these broad tumour stromal cells have been comprehensively reviewed by Xu and colleagues [14].

**Fig. 1:**
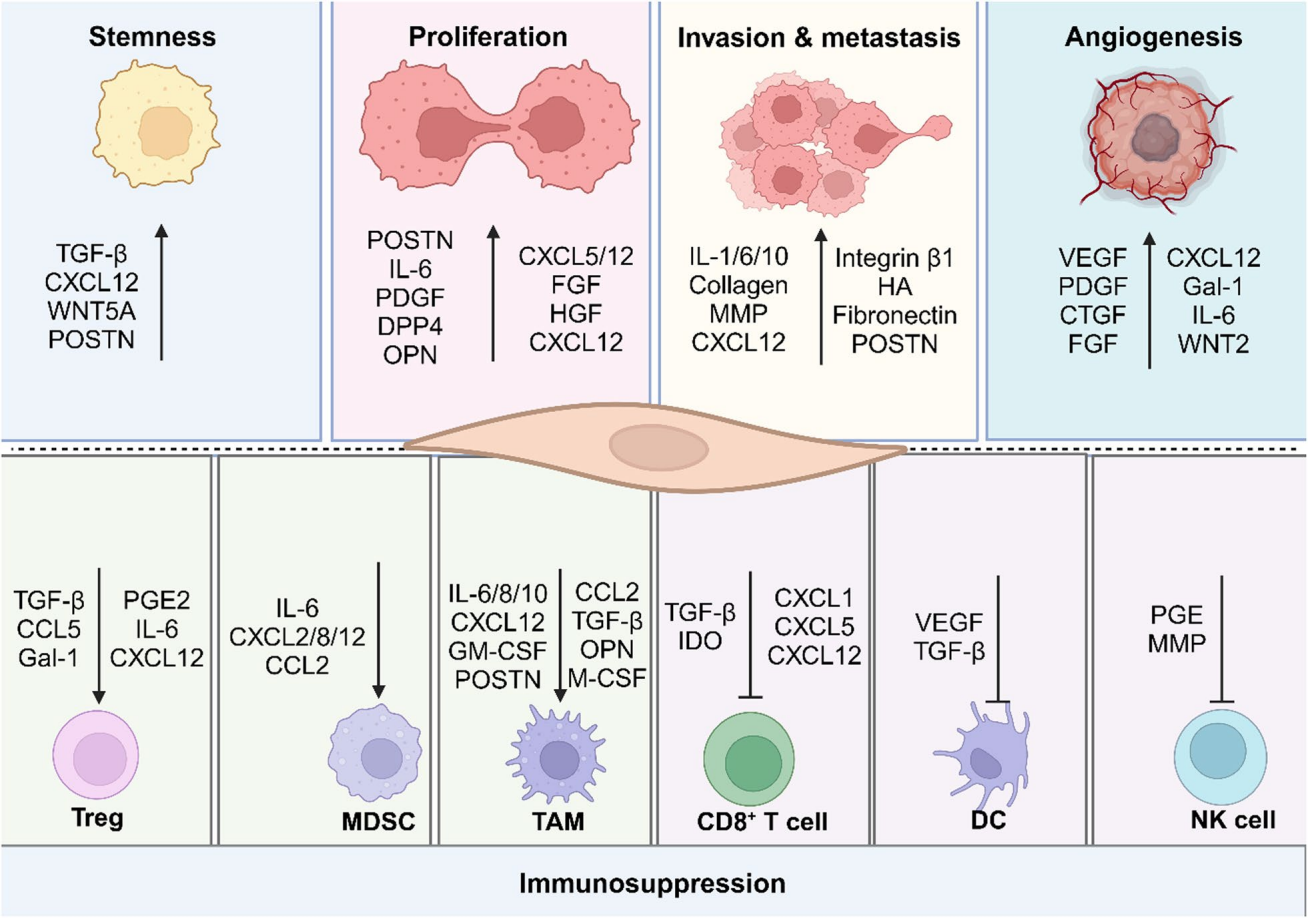
CAFs regulate cancer development and progression through interactions and communication with various cell types within the tumour microenvironment by secreting a range of factors. Treg: regulatory T cell; MDSC: myeloid-derived suppressor cell; TAM: tumour-associated macrophage; DC: dendritic cells; NK cell: natural killer cell. The figure was generated using BioRender

CAFs, a subtype of activated fibroblasts, are the most abundant and prominent cell population within the tumour stroma. Research into CAFs has surged over the past decade, greatly advancing our understanding of their roles in the TME and potential as therapeutic targets. CAFs have been shown to play multiple essential roles in cancer initiation, progression, and metastasis, through interaction and communication with cancer cells or regulating extracellular matrix (ECM) remodelling and immune cell infiltration (Fig. 1) [15–22]. In this review, we provide a summary of markers for identifying CAFs and their subtypes. Importantly, we conduct a comprehensive review of both completed and ongoing clinical trials associated with CAF-targeted therapies. We also discuss the existing challenges in CAF-related studies and proposes a direction for future research.

## CAF-related markers

Fibroblasts have long presented a challenge to define, given the absence of reliable markers specific to their cell lineage. Originating from the mesoderm during embryonic development, they share this mesenchymal lineage with adipocytes and bone cells including osteoblasts and chondrocytes. However, the few proteins that may infer a cell being a fibroblast are neither exclusive to fibroblasts alone nor uniformly present across all fibroblast subtypes [17, 23, 24]. Consequently, cells labelled as fibroblasts were often defined through negative selection—those lacking markers for epithelial, endothelial, and inflammatory cells are presumed to be fibroblasts. In addition to the absence of such markers, the morphology and location of fibroblasts are instrumental to identify them in the past. Fibroblasts exhibit an elongated, spindle-like, fusiform phenotype, characterised by a tapered structure at both ends and extended processes. Recent advancement in single-cell RNA sequencing (scRNA-seq) and imaging techniques have identified several proteins highly expressed in fibroblasts within the TME, serving as positive markers for identifying CAFs [25]. It is important to note, however, that these markers may not be universally expressed by all CAFs across different cancer types. The heterogeneity and plasticity of CAFs play a critical role in their diverse functions within tumours. CAFs can be derived or transformed from various cell types such as mesenchymal cells, normal fibroblasts, quiescent stellate cells in the pancreas and liver. Efforts to identify CAFs within the tumour stroma have led to identification and application of various CAF-related markers. These markers have diverse roles in host, CAFs, and cancer cells as shown in preclinical studies (Table 1).

**Table 1:** General CAF-related markers and their phenotypes upon target deficiency in cancer

Markers	Deficiency in host	Deficiency in CAFs	Deficiency in cancer cells	Refs.
FAP	Tumorigenesis ↓ Tumour growth ↓ Metastasis ↓ Apoptosis ↑	Tumour growth ↓ Metastasis ↓	Tumour growth ↓ Metastasis ↓	[23, 41–56, 204–207]
α-SMA	–	–	Migration ↓ Invasion ↓	[57–65, 67, 68, 208]
PDGFRα/β	–	–	Proliferation ↓ Cell death ↑	[77–85, 87–93]
Vimentin	Tumour growth ↓ Invasion ↓ Metastasis ↓	Invasion ↓	Motility ↓	[98–103, 209]
PDPN	Tumour growth ↓ Lymphatic metastasis ↓	Invasion ↓	Proliferation ↓ Migration ↓ Invasion ↓	[107, 110–112, 114–118, 210]
FSP-1	Tumorigenesis ↓	Stemness ↓	Invasion ↓ Metastasis ↓ Ferroptosis ↑	[126–131, 211]
TN-C	Immune cell infiltration ↑ Tumorigenesis ↓ Tumour growth ↓ Metastasis ↓	Angiogenesis ↓	Migration ↓ Invasion ↓ Immunosuppression ↓	[133, 141–145, 147]
POSTN	Tumorigenesis ↓ Immunosuppression ↓	Tumour growth ↓ Invasion ↓	Tumour growth ↓	[151, 153, 155–166]
Gal-1	Tumour growth ↓	Progression ↓ Migration ↓ Invasion ↓	Tumour growth ↓ Migration ↓ Invasion ↓	[168–178]
CAV1	Angiogenesis ↑ Tumour growth ↑	Tumour growth ↑ Migration ↑ Chemoresistance↑	Tumour growth ↓ Proliferation ↓ Migration ↓ Invasion ↓	[181–190]
Ephs/ ephrins	Angiogenesis ↓ Tumour growth ↓ Fibrosis ↓ Immunosuppression↑	Invasion ↓ Metastasis ↓ Fibrosis ↓	Tumour growth ↓↑ Proliferation ↓↑ Migration ↓↑ Invasion ↓↑	[191–203]

### Fibroblast activation protein (FAP)

FAP, a membrane-bound serine protease belonging to the dipeptidyl peptidase (DPP) family, is commonly expressed by CAFs and certain tumour cells such as sarcoma [26–28]. Additionally, FAP expression has been observed in fibroblasts participating in wound healing and chronic inflammatory conditions such as arthritis and cirrhosis [29–32]. The substantial upregulation of FAP is considered a biomarker for CAFs [33, 34], with potential as a unfavourable prognosis biomarker for various cancers [35–38]. However, contradictory findings suggest that high FAP expression may correlate with a better prognosis in some patients [39, 40].

High FAP expression has been correlated with enhanced tumour growth and metastatic potential [41–44]. FAP-positive (FAP^+^) CAFs produce ECM proteins that contribute to migration of tumour cells [45, 46]. In a transgenic mouse model, depletion of FAP-expressing cells led to rapid hypoxic necrosis mediated by interferon-gamma (IFN-γ) and tumour necrosis factor-alpha (TNF-α), both associated with CD8^+^ T cell-dependent tumour cell killing [47]. FAP^+^ CAF-induced immune suppression could also be mediated through the CXCL12-CXCR4 axis, and inhibition of CXCR4 resulted in eradication of cancer cells by increasing intratumoral CD8^+^ T cells [48]. Further insight into the role of FAP in the immunosuppressive TME was revealed by a murine liver tumour model, demonstrating that FAP^+^ CAFs mediate immunosuppression through STAT3-CCL2 signalling and recruitment of MDSCs [49]. Tumorigenesis and tumour growth were reduced in FAP knockout (Fap^−/−^) mice in both lung and colon cancer models [50]. Global FAP knockout delayed the onset of pancreatic tumours, increased tumour necrosis, impeded metastasis, and prolonged mice survival in the KPC (LSL-Kras^G12D/+^;LSL-Trp53^R172H/+^;Pdx-1-Cre) pancreatic cancer model [43]. Consistently, silencing FAP in CAFs was associated with impaired tumour-promoting effects in preclinical studies [51–54]. Interestingly, knockdown of FAP in cancer cells also led to reduced cell proliferation, invasion, and metastasis in oral squamous cell carcinoma (OSCC) and prostate cancer [55, 56]. These findings suggest that FAP may serve as a promising therapeutic target, in addition to its role as a CAF marker.

### α-Smooth muscle actin (α-SMA)

α-SMA is another frequently used marker for activated fibroblasts. High expression of α-SMA in CAFs was associated with poor prognosis of cancer patients [57–59]. CAFs with high α-SMA expression can stimulate growth of luminal breast cancer cells, primarily through the secretion of osteopontin (OPN) [57]. Tumours harbouring CAFs with elevated α-SMA expression exhibited high metastatic potential [58]. Conversely, CAFs expressing low levels of α-SMA suppressed self-renewal and growth of stem-like cancer cells through the signalling molecule bone morphogenetic protein 4 (BMP4) [60]. The α-SMA^+^ CAFs can promote the generation and proliferation of CD44^+^CD24^−^ breast cancer stem cells by secreting CXCL12 that activates CXCR4 on cancer cells [61]. Conditioned medium from α-SMA^+^ CAFs enhanced tumorigenicity in a co-culture assay of hepatocellular carcinoma (HCC) [62]. This effect was attributed to α-SMA^+^ CAF-derived hepatocyte growth factor (HGF), regulating the c-Met/FRA1/HEY1 signalling pathway in HCC cells [62]. Additionally, α-SMA^+^ CAFs secret a range of cytokines, such as M-CSF, IL-6, IL-8, IL-10, TGF-β, and CCL-2, inducing macrophage differentiation and M2 polarization that contributes to immunosuppressive TME [63–65]. These cytokines can also activate STAT3-PDL1 signalling in neutrophils [66], further contributing to the establishment of a suppressive TME. Although the tumour-promoting roles of α-SMA + CAFs have been demonstrated in many cancers, they appear to have the opposite effect in pancreatic cancer. In a pancreatic cancer animal model, the deletion of α-SMA + CAFs led to an increase in CD4 + Foxp3 + regulatory T cells within the tumours, resulting in accelerated tumour growth [67]. This may be due to the tumour-restricting role of the stroma, which acts as a physical barrier to limit the growth of pancreatic cancer cells and the infiltration of tumour-supporting immune cells. In fact, deletion of Sonic hedgehog (SHH) in a pancreatic ductal adenocarcinoma (PDAC) model also reduced stromal content and led to increased tumour growth [68]. These findings suggest that while eliminating α-SMA + CAFs could be a promising strategy to inhibit tumour growth in many cancer types, it should be carefully evaluated when treating pancreatic cancer.

### Platelet-derived growth factor receptor α/β (PDGFRα/β)

PDGFRα/β, a tyrosine kinase receptor, functions through the formation of homodimers (αα or ββ) or heterodimers (αβ), each exhibiting distinct interactions with PDGF ligand dimers, ultimately leading to activation of various signalling pathways [69–71]. PDGFR signalling plays a crucial role in development of organs, such as lung and kidney [72–74]. As a less specific marker for CAFs, PDGFRα/β is also expressed in normal fibroblasts, smooth muscle cells, and pericytes [75, 76]. High expression of PDGFRβ in tumour stroma was associated with large tumour size, advanced stage, and high vessel density in prostate cancer [77]. Elevated levels of PDGFRβ were associated with an increased risk of recurrence in breast and colorectal cancers [78, 79]. However, in patients with epithelial ovarian cancer, high PDGFRα/β expression in both tumour and stromal cells did not show prognostic significance [80].

Increased PDGFRα/β activity was observed in sarcoma cancer stem-like cells, promoting migration, invasion, and chemoresistance [81]. PDGFRα can interact with integrin α5β1 to promote cell contraction and reorganization of the ECM, resulting in directional migration of prostate and pancreatic cancer cells [82]. Integrin α11 also binds to PDGFRβ on CAFs, leading to increased invasion of breast cancer cells [83]. PDGFRβ^+^ CAFs, when stimulated by PDGF, can enhance migration and invasion of co-cultured colorectal cancer cells in a stanniocalcin-1-dependent manner [84]. By interacting with TGFβR, PDGFRβ can induce differentiation of MSCs into CAFs [85]. In a pancreatic cancer mouse model, PDGFRα^+^ CAFs accelerated tumour proliferation, in contrast to normal pancreatic fibroblasts that impeded tumour progression. Further categorization of PDGFRα^+^ CAFs revealed that PDGFRα^+^/SAA3 (Serum Amyloid A3)^+^ CAFs could enhance PDAC progression, whereas PDGFRα^+^ CAFs without SAA expression suppress tumour growth, attributed to Mpp6 overexpression [86].

The immunomodulatory effects of PDGFRα/β^+^ CAFs have also been well-documented in several studies. PDGFRα^+^ CAFs secrete Chitinase 3-like 1 to induce macrophage recruitment and M2 polarization in breast cancer [87]. In a co-culture assay, T cells, in the presence of PDGFRα/β^+^PDPN (podoplanin) ^+^ CAFs, exhibited low cytotoxicity towards co-cultured tumour cells [88]. The reduced T cell cytotoxicity could be resulted from increased apoptosis of FAS-expressing CD8^+^ T cells, a process mediated through the expression of FAS ligand and programmed death-ligand 2 (PD-L2) by CAFs [88]. Interestingly, high PDGFRα expression in CAFs was also associated with increased immune infiltration, potent T cell cytotoxicity, and prolonged survival in PDAC [89], highlighting the complex roles of PDGFRα/β in different contexts. Therefore, PDGFRα/β-targeted monotherapy may not be suitable for treating PDAC. In addition to its interesting roles in CAFs, PDGFR also plays a direct role in cancer cells. Knockdown or knockout of PDGFRA in gastrointestinal (GI) and glioblastoma (GBM) cancer cells suppressed tumour proliferation [90–92]. In BRCA1-deficient breast cancer cells, deletion of PDGFRβ promoted cell death and inhibited tumorigenesis [93]. PDGFRα/β could therefore serve as promising targets for direct anti-cancer therapy in treating these cancers.

### Vimentin

Vimentin, a type III intermediate filament protein, serves as a major component of the cytoskeleton in non-epithelial cells, particularly mesenchymal cells. While high vimentin expression was observed in CAFs, normal fibroblasts (NFs) also exhibited similar levels of vimentin [94]. Presence of α-SMA^−^Vimentin^+^ CAFs was associated with poor survival in PDAC patients [95], and high vimentin expression in tumour stroma was linked to high malignant potential and disease recurrence in colorectal cancer (CRC) patients [96]. Interestingly, another study found that low vimentin expression in stroma and high vimentin expression in cancer cells was associated with prolonged overall survival (OS) in patients with ovarian tumours [97].

Vimentin plays diverse roles in EMT, focal adhesion, migration, invasion, and metastasis of cancer cells [98, 99], but knowledge on the function of vimentin in CAFs is limited. In a preclinical study employing a Cre-dependent *LSL-Kras*^*G12D*^*/Lkb1*^*fl/fl*^ lung cancer model, vimentin was expressed in CAFs surrounding collective invasion packs of epithelial tumour cells, and whole-body vimentin knockout led to a reduction of invasion packs [100]. In a non-small cell lung cancer (NSCLC) model induced by *LSL-Kras*^*G12D*^/*Tp53*^*fl/fl*^, whole-body knockout of vimentin attenuated cancer-associated cachexia symptoms, inhibited tumour growth, and led to improved survival [101]. Vimentin also plays a direct role in cancer cells, as demonstrated by the observation of reduced cell motility in cancer cells upon vimentin knockdown [102, 103]. Future studies on the detailed function of Vimentin^+^ CAFs will be beneficial to understand their specific roles in cancer development.

### Podoplanin (PDPN)

Podoplanin (PDPN) is a mucin-type protein with diverse physiological and pathological functions. PDPN-deficient mice displayed defects in blood-lymphatic vascular separation, impacting proper regulation of lymph flow [104, 105]. While high PDPN expression was predominantly found in lymphatic endothelium and often utilised as a marker for lymphatic vessels [106], elevated PDPN expression has been reported in CAFs and associated with poor outcomes in various cancer types, including lung [107], breast [108], and pancreatic cancers [109]. The roles of PDPN^+^ CAFs have been explored in several studies. In a collagen invasion assay involving co-cultured cancer cells and CAFs, PDPN^+^ CAFs created invasion tracks for lung cancer cells, and knockdown of PDPN in CAFs decreased invasion of both CAFs and cancer cells [110]. However, ectopic expression of PDPN in human fibroblasts did not affect the migratory and invasive properties of co-cultured breast cancer cells [111]. PDPN^+^ CAFs showed high expression of TGF-β and were associated with CD204^+^ TAM infiltration in stage-I lung squamous cell carcinoma, leading to the immunosuppressive TME [107]. Interestingly, PDPN^+^ CAFs also exhibited a tumour-inhibitory effect by suppressing the proliferation of small cell lung cancer (SCLC) cells in a co-culture assay [112]. Another study suggested an association between PDPN^+^ CAFs and prolonged disease-free survival (DFS) in CRC patients [113]. PDPN could also act as a co-inhibitory receptor on T cells, and T cell specific PDPN conditional knockout mice exhibited delayed tumour growth [114]. Macrophage-specific PDPN conditional knockout mice showed reduced lymph angiogenesis and lymph invasion in breast cancer [115]. Knockdown of PDPN in cancer cells also resulted in reduced cell proliferation, migration, and invasion [116–118], suggesting the intricate roles of PDPN in cancer development.

### Fibroblast-specific protein 1 (FSP-1)

FSP-1, also known as S100A4, is a well-established marker for fibroblasts involved in tissue remodelling [119, 120]. Although FSP-1 is expressed in both CAFs and NFs, CAFs from cancer tissues generally exhibit more abundant FSP-1 expression than NFs from adjacent normal tissues [121]. Increased FSP-1 expression in CAFs was linked to EMT [122], and its presence was detected in inflammatory macrophages [123]. In CRC patients, high FSP-1 expression in CAFs was associated with tumour invasion [124]. Intriguingly, tumoral FSP-1 positivity and stromal FSP-1 negativity was correlated to short DFS and OS in patients with invasive lobular carcinoma [125].

FSP-1^+^ CAFs promote tumour metastasis by secreting factors such as VEGF-A and Tenascin-C, establishing an angiogenic microenvironment at metastatic sites and providing protection from apoptosis [126]. In addition, monocyte chemotactic protein-1 derived from FSP-1^+^ CAFs increased monocyte recruitment and inflammatory responses in a skin tumour model [127]. Mice with FSP-1 deficiency had decreased tumour incidence, and co-injection of FSP-1^+^ CAFs with mouse mammary carcinoma cells partially restored tumour development and metastasis [128]. However, depletion of FSP-1^+^ stromal cells did not prevent the development of hepatocellular carcinoma (HCC), although it reduced the stemness phenotype of tumours [129]. Head and neck squamous cell carcinoma (HNSCC) cells with FSP-1 knockdown exhibited reduced expression of matrix metalloproteinase 3 (MMP3), resulting in decreased invasiveness and metastasis in vivo [130]. The loss of FSP-1 in cancer cells resulted in increased ferroptosis and cell death upon the treatment of ferroptosis-inducing agent [131]. These findings suggest that FSP-1 could be a promising anti-tumour target, given its tumour-promoting roles in both CAFs and cancer cells.

### Tenascin-C (TN-C)

TN-C, a glycoprotein interacting with ECM molecules like fibronectin [132], is abundantly expressed by CAFs and solid malignant tumours [133–135]. TN-C expression in the stroma of prostate cancer showed correlation with the expression of other CAF markers, including FSP-1, α-SMA, and vimentin [135]. In pancreatic cancer, TN-C in CAFs can enhance epithelial-to-mesenchymal transition and is associated with resistance to immune checkpoint inhibitors in patients [136]. High tumoral TN-C expression could be associated with tumour progression, metastasis, and poor prognosis in different cancer types [137–140]. TN-C produced by CAFs promoted metastasis of colon cancer cells in response to TGF-β signalling [133]. In an osteosarcoma xenograft model, TN-C contributed to lung metastasis by interacting with its receptor integrin α9β1 [141]. TN-C also promoted immune suppression by immobilising infiltrated T lymphocytes through chemokine (C-X-C motif) ligand 12 (CXCL12) signalling [142]. Moreover, TN-C increased infiltration of Treg cells, and ablation of TN-C inhibited immune-suppressive stromal properties in an OSCC model [143]. TN-C knockout mice exhibited increased immune cell infiltration and reduced tumorigenesis, tumour size, and tumour metastasis compared to wild type mice [143, 144]. Knockdown of TN-C in CAFs led to increased endothelial tubulogenesis of glioblastoma [145], and TN-C knockout in tumour cells reduces lymphoid immune suppression, migration, and invasion of osteosarcoma and OSCC [140, 143]. A recent study found that reducing TN-C expression in cancer cells can enhance the efficacy of inhibitors targeting the ErbB3, PI3K-AKT, Ras, and MAPK signalling pathways in oesophageal squamous cell carcinoma [146]. More in-depth exploration of TN-C roles in cancer has been reviewed by others [147, 148].

### Periostin (POSTN)

POSTN is a secreted cell adhesion glycoprotein that serves as a ligand for integrins αVβ3 and αVβ5. High expression of POSTN in CAFs was associated with poor prognosis in various cancers, including breast [149], cervical [150], CRC [151, 152], oesophageal cancers (EAC) [153], and PDAC [154]. The colony number and spheroid size of CRC were significantly larger when co-cultured with *Postn*^+*/*+^ fibroblasts than when co-cultured with POSTN knockdown or knockout fibroblasts [151, 155]. When binding to integrin αVβ3, CAF-derived POSTN can activate PI3K/AKT signalling pathway, promoting EMT, migration and invasion of ovarian cancers and EAC [153, 156]. The ERK pathway can also be activated by CAF-derived POSTN, leading to enhanced proliferation, migration, and EMT of NSCLC and gastric cancer cells [157, 158]. Downregulating POSTN in the TME of PDAC reduced proliferation, metastasis, and clonality of PDAC cells [159]. POSTN also showed interaction with protein tyrosine kinase 7 in HNSCC to promote cancer stemness [160]. Through bindings to integrins αVβ3 and αVβ5, POSTN can activate the ERK/NF-κB signalling pathway in ovarian cancer cells, leading to increased expression of cytokines that promote macrophage mobility and polarization toward the M2 phenotype [161]. POSTN also induced expression of Programmed Cell Death Protein 1 (PD-1) on TAMs through integrin-ILK-NF-κB signalling, and PD-1^+^ TAMs, in turn, produced IL-6 and IFN-γ, leading to induction of Programmed Cell Death Ligand 1 (PD-L1) expression on CRC cells [162]. POSTN knockout (*Postn*^*−/−*^) mice exhibited reduced infiltration of PD-1-positive TAMs in CRC tumours [162] and displayed a lower tumorigenic potential [163, 164]. Intriguingly, *Postn*^*−/−*^ mice demonstrated impaired capsule formation and enhanced tumour growth in another study [165]. Tumoural POSTN may also contribute to tumour growth and knockdown of POSTN in lung cancer cells repressed tumour growth in vivo [166]. These studies suggest the diverse roles of POSTN, underscoring its potential as a therapeutic target.

### Galectin-1 (Gal-1)

Gal-1, a member β-galactoside-binding protein family, is ubiquitously expressed both intracellularly and extracellularly, despite lacking a secretion signal peptide [167]. Gal-1 plays a crucial role in cell–cell and cell–matrix adhesion in the TME, and CAF-derived Gal-1 induced metastasis, EMT, and angiogenesis in gastric cancer [168, 169]. TGF-β secreted from gastric cancer cells could transform NFs into CAFs by upregulating Gal-1 and α-SMA expression in fibroblasts [170]. Elevated Gal-1 expression in CAFs contributed to adaptive resistance to tyrosine kinase inhibitors (TKIs) of anaplastic lymphoma kinase in NSCLC [171]. Knockdown of Gal-1 in CAFs reduced the expression of monocyte chemoattractant protein-1 (MCP-1) and inhibited the progression of OSCC in vivo [172]. Gal-1 knockdown in CAFs also reduced migration and invasion of breast cancer cells by downregulating MMP9 expression [173]. Interestingly, tumour-derived Gal-1 increased frequency of CD4^+^CD25^+^Foxp3^+^ Treg cells in breast cancer [174], leading to an immunosuppressive TME. In addition, knockdown of Gal-1 in cancer cells reduced migration, invasion, and tumour growth [175, 176]. Gal-1 deficient mice exhibited impaired tumour growth due to inadequate tumour angiogenesis and a less immunosuppressive TME [177, 178]. In summary, Gal-1 serves as a marker for CAFs and presents itself as a promising target for cancer treatment.

### Caveolin 1 (CAV1)

CAV1, a scaffolding protein crucial for the formation of caveolae, is involved in processes like endocytosis and receptor internalization [179], exhibiting both tumour-suppressive and tumour-promoting properties [180]. In the transition of NFs into CAFs, CAV1 expression was significantly downregulated, making it a negative marker for CAFs [181]. Low expression of CAV1 in CAFs was an independent predictor of poor prognosis in gastric cancer patients [182]. CAFs with reduced CAV1 expression exhibited an enhanced glycolytic phenotype, promoting migration and progression of PDAC [183]. Knockdown of CAV1 in fibroblasts not only promoted tumour growth but also increased chemoresistance in PDAC and HCC [184, 185]. CAV1 deficiency in CAFs also led to increased production and secretion of pro-inflammatory and tumour-enhancing cytokines, contributing to proliferation and invasion of gastric cancer cells [186]. Moreover, CAV1-deficient mice exhibited increased tumour permeability, angiogenesis, and growth in different tumour models [187, 188]. Interestingly, knockdown of CAV1 in cancer cells resulted in attenuated tumour growth, decreased proliferation, and impaired migration and invasion [189, 190], suggesting a tumour-promoting role of CAV1 in cancer cells. However, the detailed mechanisms of how CAV1 regulates cancer cells remain to be investigated.

### Ephs/ephrins

Eph receptors and their membrane bound ephrin ligands control cell–cell interactions during development, including tissue boundary formation and patterning of the neural and vascular systems, and are often up-regulated in tumours and the TME, including on CAFs [191, 192]. Several Eph receptors have been identified as elevated in stromal cells from human gastric tumours compared to those from normal tissues, and expression of EphA2 was associated with poor prognosis [193]. In co-culture assays including CAFs and cancer cells, tyrosine phosphorylation of EphA2 on CAFs was increased, which led to enhanced invasiveness of cancer cells [194, 195]. Similarly, ephrin-B on fibroblasts was found to increase invasiveness of EphB3/4-expressing prostate cancer cells [196]. EphA3 was identified to be widely expressed in the stroma of diverse cancer types, present on MSCs and specific CAF subtypes in human and mouse tumours [197, 198]. Antibody targeting [197] or knock-down of TME-expressed EphA3 [198] decreased angiogenesis and tumour growth. In breast cancer, EphA3 was identified on both cancer cells (upregulated by RAGE signalling), and on CAFs, and its activity promoted invasion, which was blocked by a specific EphA3 inhibitor [199]. Ephrin-A5 expression was identified on pancreatic CAFs and thought to mediate interaction with EphA receptors on cancer cells, as well as on other CAFs, and to promote collagen synthesis [200]. Ephrin-B2 expressed on lung and pancreatic myofibroblasts was found to be shed by the transmembrane metalloprotease ADAM10, leading to fibroblast activation and fibrosis, and inducing EphB4 signalling in pancreatic cancer cells [201, 202]. Multiple ephrin-Bs were similarly found to be elevated in prostate CAFs and to promote CAF activation, cancer cell proliferation, and tumorigenicity in vivo [203]. Thus, while Eph and ephrin expression in tumour cells can have both tumour suppressive and promoting roles [191], their expression in CAFs appears exclusively tumour-promoting.

## CAF subtypes

**Fig. 2:**
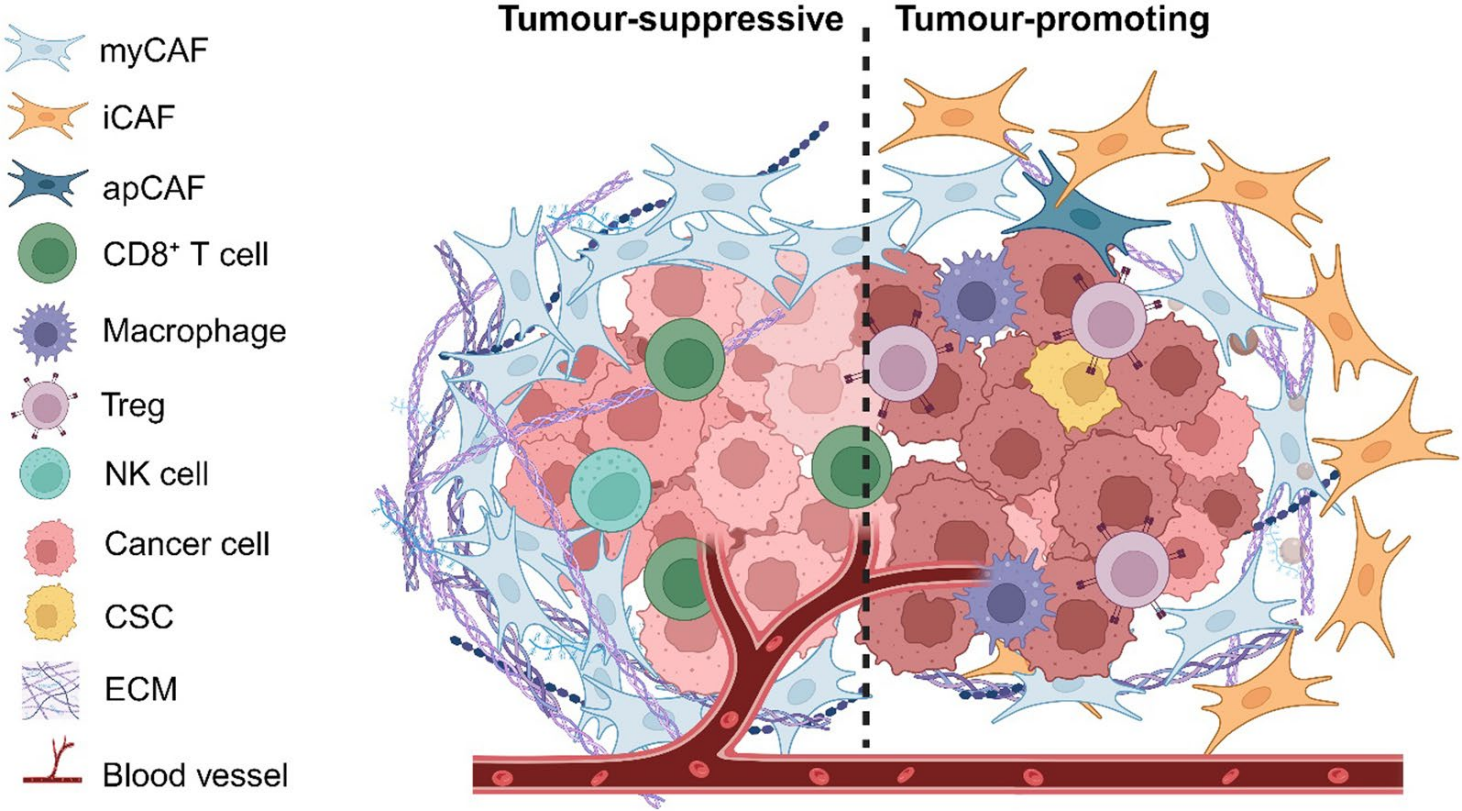
Tumour-suppressive and tumour-promoting roles of CAF subtypes in TME. CAF: cancer-associated fibroblast; myCAF: myofibroblastic CAF; iCAF: inflammatory CAF; apCAF: antigen-presenting CAF; Treg: regulatory T cell; NK cell: natural killer cell; CSC: cancer stem cell; ECM: extracellular matrix. The figure was generated using BioRender

The heterogeneity of CAFs is supported by several key findings. Firstly, the molecular markers employed for CAF identification are diverse and lack complete specificity, often failing to encompass the entire CAF population. Minimal co-localisation of commonly used CAF markers, such as FSP1, αSMA, and PDGFRβ, was observed in tumour stroma of pancreatic and breast cancer mouse models, highlighting the inability of these markers to represent all CAFs in isolation [212]. Secondly, attempts to antagonise CAFs to reduce tumour burden have yielded contradictory outcomes, emphasising the intricate roles of CAFs, which are potentially associated to their heterogeneity. The growing recognition of CAF heterogeneity has encouraged extensive investigations on CAF subtypes that play tumour-suppressive and tumour-promoting roles in the TME (Fig. 2). The advance of scRNA-seq technology has also facilitated the identification and stratification of CAF subtypes in different cancers (Table 2). It is important to note, however, that there are no definitive factors to clearly stratify the pro-tumour or anti-tumour functions of CAFs.

**Table 2:** Major CAF subtypes in different cancers

Subtypes	Markers/Expression signatures	Cancers	Refs.
myCAFs	α-SMA^high^IL-6^low^	PDAC	[213]
myCAFs	TNC, α-SMA, TGF-β1, SERPINE2	Breast	[220]
myCAFs	FAP, CD90, α-SMA, PDPN, COL1A1, COL1A2	Breast	[217]
myCAFs	α-SMA, TAGLN, MYL9, IGFBP3, TNC, TGF-β1, TGF-β2, CTGF	Breast	[218]
myCAFs	α-SMA, TAGLN	Gastric/ovarian	[221]
myCAFs	α-SMA, TAGLN	CRC	[222]
myCAFs	COL1A1, COL1A2, FAP, PDPN	CRC	[223]
myCAFs	MMP11, WNT5A	SCC	[228]
myCAFs	α-SMA, TAGLN, VIM, FN1, MMP11, COL1A1, COL3A1, COL15A1, COL16A1, FAP	ccRCC	[229]
myCAFs	α-SMA, TAGLN, MYL9, TPM, COL1A1, COL1A2	Prostate	[224]
myCAFs	α-SMA, COL1A1, COL8A1, COL15A1, CRLF1, FBN2, SERPINF1	Liver	[230]
myCAFs	TPM1, TPM2, MYL9, TAGLN, POSTN	Gastric	[227]
iCAFs	FAP^+^α-SMA^low^IL-6^high^	PDAC	[213]
iCAFs	CD34, CD26, CXCL12, FSP-1, C3, DPP4	Breast	[220]
iCAFs	CXCL12, CD34	Breast	[217]
iCAFs	Ly6C1, CLEC3B, HAS1, DPT, COL14A1, IL6, IL33, CXCL1, CXCL12, CCL7	Breast	[218]
iCAFs	PDGFRα, CFD, CXCL12	Gastric/ovarian	[221]
iCAFs	ICAM1, PDPN	CRC	[222]
iCAFs	CXCL12	CRC	[223]
iCAFs	C3, IGF1	SCC	[228]
iCAFs	CXCX12, IGF1, C3, C7, CFD, CFH,	Prostate	[224]
iCAFs	LRAT, RELN, RGS5	Liver	[230]
iCAFs	IL6, IL11, IL24, CXCL1, CXCL2, CXCL5, CXCL6, MMP1, MMP3, MMP10	Gastric	[238]
iCAFs	PDGFRα, IL6, CXCXL1, CXCL2, CXCL12, CXCL14,	Bladder	[242]
apCAFs	MHC-II genes, CD74, SAA3, SLPI	PDAC	[213]
apCAFs	MHC-II genes, CD74, FSP1, KRT8, KRT18	Breast	[218]
apCAFs	MHC-II genes, CD74, IL-8, POSTN	ccRCC	[229]
apCAFs	MHC-II genes, CD74	Prostate	[224]
apCAFs	MHC-II genes	Lung	[246]

myCAFs: myofibroblastic CAFs; iCAFs: inflammatory CAFs; apCAFs: antigen-presenting CAFs. α-SMA: α-Smooth muscle actin; IL-6/11/24/33: interleukin 6/11/24/33; TNC: tenascin C; TGF-β1/2: transforming Growth Factor-beta 1/2; SERPINE2: Serpin Family E Member 2; FAP: fibroblast activation protein alpha; PDPN: podoplanin; COL1A1/2, collagen type I alpha 1/2; TAGLN: transgelin; MYL9: myosin light chain 9; IGFBP3: insulin like growth factor binding protein 3; CTGF: connective tissue growth factor; MMP1/3/10/11: matrix metalloproteinase 1/3/10/11; WNT5A: Wnt Family Member 5A; VIM: vimentin; FN1: fibronectin1; COL3/8/14/15/16A1: collagen type 3/8/14/15/16 alpha 1; TPM1/2, tropomyosin 1/2; CRLF1, cytokine receptor-like factor 1; FBN2: fibrillin 2; SERPINF1 Serpin Family F Member 1; POSTN: periostin; CXCL: C-X-C motif chemokine ligand; FSP-1: fibroblast-specific protein-1; C3/7: complement component 3/7; DPP4: dipeptidyl peptidase 4; Ly6C1: lymphocyte antigen 6 family member C1; CLEC3B: C-type lectin domain family 3 member B; HAS1: hyaluronan synthase 1; DPT: dermatopontin; CCL7: C-C motif chemokine ligand 7; PDGFRα: platelet-derived growth factor receptor alpha; CFD/H: complement factor D/H; ICAM1: Intercellular Adhesion Molecule 1; IGF1: Insulin Like Growth Factor 1; LRAT: lecithin-retinol acyltransferase; RELN: reelin; RGS5: regulator of G protein signalling 5; MHC-II: major histocompatibility complex II; SAA3: serum amyloid A3; SLPI: secretory leukocyte peptidase inhibitor; KRT8/18: keratin 8/18

### Myofibroblastic CAFs (myCAFs)

Öhlund et al. firstly identified two distinct subpopulations of CAFs, including myCAFs and inflammatory CAFs (iCAFs) in pancreatic cancer [213]. The myCAFs, characterised by high α-SMA expression and low IL-6 expression (α-SMA^high^IL -6^low^) phenotype, were in close proximity to neoplastic cells, forming a structural ring surrounding clusters of cancer cells. These myCAFs exhibited an upregulation of TGF-β response targets such as CTGF and COL1A1. A subsequent study found that TGF-β secreted by PDAC cells contributes to the generation of myCAFs by downregulating IL1R1 expression [214].

One distinctive feature of myCAFs is their high contractility and ability to synthesise key ECM proteins like collagens [215]. This unique trait is believed to contribute to tumour tissue stiffness, creating a physical barrier that constrain tumour growth and impact treatment efficacy. Depletion of α-SMA^+^ myCAFs in a transgenic murine PDAC model resulted in increased invasion, induction of EMT, emergence of stem-like properties, reduced overall survival (OS), and elevated presence of CD4^+^Foxp3^+^ Treg cells [67]. Absence of α-SMA^+^ myCAFs in the PDAC model also rendered tumours unresponsive to gemcitabine treatment. However, administration of anti-CTLA4 therapy showed potential in slowing disease progression and extend survival in the α-SMA^+^ myCAFs depleted model. Hedgehog (HH) signalling is generally activated in myCAFs, and depletion of SHH (a HH ligand) in PDAC tumours led to reduced stroma content but more aggressive cancer with increased vascularity [68]. These studies highlight the intricate relationship between myCAFs and tumour growth in PDAC. Although the ECM established by myCAFs may impede drug delivery, the physical barrier and stiffness could act as constraints against tumour growth. Consequently, therapeutic strategies targeting myCAFs in PDAC should be carefully considered.

Precision in therapeutic approaches targeting CAFs is crucial, as strategies focusing on CAF depletion may inadvertently lead to loss of other tumour-suppressive cells, potentially exacerbating tumour aggressiveness. Krishnamurty et al. revealed that markers like α-SMA and FAP were expressed in multiple stromal cell types, including fibroblasts and pericytes in both murine PDAC tumours and normal tissues [216]. Recently, a study demonstrated that leucine rich repeat containing 15 (LRRC15) displayed a more specific expression pattern in myCAFs, and targeted depletion of LRRC15^+^ myCAFs resulted in a substantial 70% reduction in overall PDPN^+^ CAFs, significantly attenuating PDAC tumour growth [216]. This selective depletion prompted a transformation of the remaining CAFs within PDAC tumours into a more universally fibroblast-like state. Moreover, elimination of LRRC15^+^ myCAFs enhanced the function of CD8^+^ T cells, rendering them more effective in response to anti-PD-L1 treatment. Taken together, myCAFs remain to be a promising and feasible therapeutic target for PDAC when approached with precision in target selection.

The presence of myCAFs has also been documented in breast cancer, and these myCAFs may contribute to immunosuppression and resistance to immunotherapy [217–219]. The myCAFs in breast cancer exhibited increased secretion and alignment of collagens, which could promote tumour growth and invasion [217]. A recent study suggested that myCAFs in breast cancer may originate from a specific subset of fibroblasts known as CD26^−^ NFs [220]. The roles of myCAFs in many other cancers have also been studied, revealing diverse functions in different cancer types [221–225]. In castration-resistant prostate cancer (CRPC), SPP1 (Secreted Phosphoprotein 1)^+^ myCAFs were shown to promote resistance to androgen deprivation therapy via paracrine activation of the ERK signalling pathway [226]. In lung cancer, myCAFs marked by FAP and α-SMA expression exhibited a high level of fibrillar collagens, contributing to the formation of a dense ECM that can restrict the motility of T cells [225]. In gastric cancer, a specific subtype of myCAFs characterised by IGFBP7 expression enhanced cancer cell metastasis and stemness [227]. In squamous cell carcinoma (SCC), both myCAFs and iCAFs were involved in secretion of collagens and fibronectin 1, which can interact with CD44 on SCC keratinocytes and lead to increased cancer cell proliferation and invasion by activating PI3K/AKT and Src/MAPK signalling pathways [228]. Using scRNA-Seq and spatial analysis, Davidson et al. demonstrated that myCAFs were in close proximity to and strongly interacted with mesenchymal-like clear cell renal cell carcinoma (ccRCC) within primary tumours and metastatic sites [229]. This interaction promoted cancer invasion through secretion of multiple ligands acting on cancer cells. In liver cancer, myCAFs secrete hyaluronan by overexpressing hyaluronan synthase 2 (HAS2), leading to increased tumour growth [230]. Conditional knockout of *Has2* in CAFs resulted in reduced hyaluronan production and, consequently, smaller tumour sizes in preclinical models. Interestingly, blocking tumoral CD44 receptor for hyaluronan did not inhibit cancer development, suggesting potential interactions of hyaluronan with non-tumour cells or other receptors [230]. In another study, depletion of myCAFs was found to reduce tumour growth and mortality in desmoplastic CRC and pancreatic metastasis [231]. In summary, myCAFs demonstrated both tumour-promoting and tumour-suppressive effects. Future studies should focus on developing treatments that can diminish their tumour-promoting effects while preserving tumour-suppressive functions.

### Inflammatory CAFs (iCAFs)

In PDAC, iCAFs were characterized by low α-SMA expression and high levels of inflammatory cytokines, such as IL-6 and leukemia inhibitory factor [213]. These cells exhibited a loss of myofibroblastic features and are typically situated at a distance from cancer cells [213]. Activation of the JAK/STAT pathway by tumour-derived IL-1 is recognised as a driver for the formation of iCAFs [214]. Hypoxia in the TME could also contribute to the generation of iCAFs, resulting in their enrichment in hypoxic tumour regions [232, 233]. In addition, the formation of iCAFs can be induced by IL-17A derived from a specific subpopulation of CD8^+^ T cells, known as Tc17 cells [234]. Compared to untreated patients, pancreatic cancer patients resistant to chemotherapy exhibited high levels of iCAFs in tumour stroma, indicating a role of iCAFs in chemoresistance [235]. Zhang et al. also reported an increased population of iCAFs in chemo-resistant PDAC patients following chemotherapy, while the abundance of myCAFs remained unchanged [236]. Despite the predominant pro-tumorigenic roles attributed to iCAFs in various studies, a cluster of tumour-restrictive iCAFs characterised by high expression of osteoglycin was identified [237]. In PDAC patients, iCAF-derived osteoglycin serves as a favourable prognostic biomarker for OS [237].

The pro-tumorigenic roles of iCAFs extend beyond pancreatic cancer and are reported in other cancer types. In breast cancer, iCAFs recruit myeloid cells in a CXCL12-dependent manner and enhance MMP activity, ultimately leading to increased tumour invasion [220]. The spatial distribution of iCAFs in breast cancer mirrored that observed in pancreatic cancer, positioning iCAFs relatively distal to the invasive tumour surface [217]. The abundance of iCAFs in breast tumour tissues correlated with the infiltration of Treg cells as well as dysfunction of cytotoxic T-lymphocytes [217]. Interestingly, the iCAF-like fibroblasts characterised by PDGFRβ^+^α-SMA^low^CD34^high^CD146^−^ was also abundantly detected in the surrounding ductal regions of healthy breast tissue [217]. In liver cancer, iCAFs showed high expression of HGF, promoting tumour growth via the HGF-MET axis [230]. Conditional depletion of HGF in CAFs resulted in decreased development of liver cancer, and depletion of the HGF receptor MET in hepatocytes or tumour compartments reduced tumour growth [230]. In gastric cancer, iCAFs were enriched with pro-stemness-associated pathways, including NF-κB signalling, TNF signalling, and cytokine-receptor interaction pathways, implying their involvement in cancer stemness [238]. Moreover, iCAFs showed interaction with surrounding T cells by secreting IL-6 and CXCL12, leading to establishment of a tumour-favourable microenvironment in gastric cancer [239]. In CRC, fibroblast growth factor 19 (FGF19) derived from tumour cells can induce the formation of iCAFs through the FGFR4-JAK2-STAT3 pathway [240]. These iCAFs subsequently promoted liver metastasis by increasing neutrophil infiltration and the formation of neutrophil extracellular traps in liver metastatic niches. Some iCAFs showed high expression of IL1R1 and addition of an IL-1-inhbiting antibody effectively reduced tumour spheroid growth [241]. Elevated levels of IL1R1 in CRC cancer patients were correlated with an increased expression of T cell exhaustion markers like LAG3, as well as immunoregulatory proteins such as PD-L1 and PD-L2 [241]. In bladder cancer, iCAFs express a variety of growth factors that contribute to angiogenesis, cancer cell proliferation, and chemoresistance [242]. In preclinical models of PDAC, while ablation of the tumour-restrictive α-SMA^+^ CAFs reduced survival, depletion of FAP^+^ CAFs significantly improved survival and enhanced the efficacy of immune checkpoint inhibitors (ICIs) [243]. Further research is needed to assess the efficacy of iCAF-targeted therapies.

### Antigen-presenting CAFs (apCAFs)

In addition to the two predominant CAF subtypes described above, scRNA-seq studies have identified a less common cluster of CAFs characterised by high expression of MHC-II genes and CD74 [244]. These distinct CAFs were designated as apCAFs due to their unique ability to activate CD4^+^ T cells in an antigen-specific manner. It should be noted apCAFs are not abundant and only sporadically detected in most cancers. In pancreatic cancer, apCAFs may originate from mesothelial cells, a transformation induced by IL-1 and TGF-β. These apCAFs can directly interact with naive CD4^+^ T cells, resulting in formation of Treg cells [245]. The use of a blocking antibody targeting mesothelin, a marker associated with mesothelial cells, can effectively inhibit the transition of mesothelial cells into apCAFs, leading to reduced Treg cells and attenuated tumour growth [245]. However, in lung cancer, apCAFs showed a distinct role by directly activating T cell receptors on adjacent effector CD4^+^ T cells and producing C1q to rescue these T cells from apoptosis [246]. Deletion of MHC-II to reduce apCAFs led to increased tumour burden, reduced survival rates, and fewer infiltrated T cells, implying a tumour-suppressive effect of apCAFs in lung cancer [246]. Due to the low abundance of apCAFs, their roles in cancer development have not been extensively studied. More studies will be required to examine their therapeutic potential.

### Other CAF subtypes

Despite the initial classification of myCAF, iCAF, and apCAF subtypes in pancreatic cancer, researchers have also identified different CAF subtypes. In PDAC, CAFs expressing Meflin were associated with a better response to chemotherapy, and inducing Meflin expression in CAFs could enhance sensitivity of PDAC tumours to gemcitabine [247]. These Meflin^+^ CAFs were referred as "rCAFs" due to their capacity to restrain tumour growth. Further investigations revealed that Meflin directly inhibits lysyl oxidase, an enzyme responsible for crosslinking collagen and elastin, contributing to tissue stiffness and increased interstitial pressure [247]. The advancements in scRNA-seq technology have facilitated a more comprehensive analysis of CAF subtypes, especially in cases where a sufficient number of stromal cells are available. For instance, Cords et al. conducted an in-depth analysis of CAF subtypes by employing scRNA-seq on over 16,000 stromal cells obtained from 14 breast cancer patients [248], leading to identification of nine distinct CAF subtypes, each characterized by unique molecular signatures and functions. In cribriform prostate cancer, a specific subtype of CAFs characterized by the CTHRC1^+^ASPN^+^FAP^+^ENG^+^ signature was referred to as "CAFÉ CAFs", which was associated with an immunosuppressive TME [249]. In lung cancer, a subpopulation CAFs characterised by ZIP1^+^FSP1^+^CX43^high^, known as "zCAFs", can absorb and transfer Zn^2+^ to neighbouring cancer cells via gap junctions, leading to chemoresistance [250]. Another subtype of CAFs with MYH11^+^α-SMA^+^CD34^+^FAP^−^ADH1B^−^ signature was associated with reduced infiltration of CD3^+^ and CD8^+^ T cells, contributing to immune exclusion within tumour nests [225]. Several other CAF subtypes have been characterized in different cancers, and these subtypes have been reviewed extensively by other researchers [251–253].

### Normal fibroblasts (NFs)

NFs are widely distributed in many healthy organs and tissues, where they play crucial roles in development, homeostasis, injury repair, and normal signalling. NFs secrete structural macromolecules, such as collagen, contributing to the synthesis, remodelling, and maintenance of the ECM [254]. In addition, NFs serve as a rich source of signalling molecules, including growth factors, cytokines, and chemokines, which act on other cells to regulate development and other biological processes [255]. In response to tissue damage, NFs can rapidly expand to produce more ECM-secreting fibroblasts that are critical to tissue synthesis, as well as myofibroblasts with high expression of contractile proteins such as α-SMA [256]. Tissue-specific fibroblasts have been found organs such as skin, lung, colon, skeletal muscle, and heart, where they support organ development and homeostasis [257, 258]. Various molecular markers have been reported to identify NFs, with widely used pan-fibroblast markers including CD90, PDGFRα/β, vimentin, and collagens [259]. Markers and genes enriched in specific NF subtypes have been reviewed by elsewhere [259–261]. It is important to note, however, that some NF markers are also expressed by other cell types, such as the high PDGFRβ expression found in pericytes and smooth muscle cells [262]. Given that CAFs can originate from NFs, there is substantial overlap in markers between these two fibroblast types, making it critical to approach CAF analysis with caution to avoid NF contamination.

## Directly targeting CAFs

**Fig. 3:**
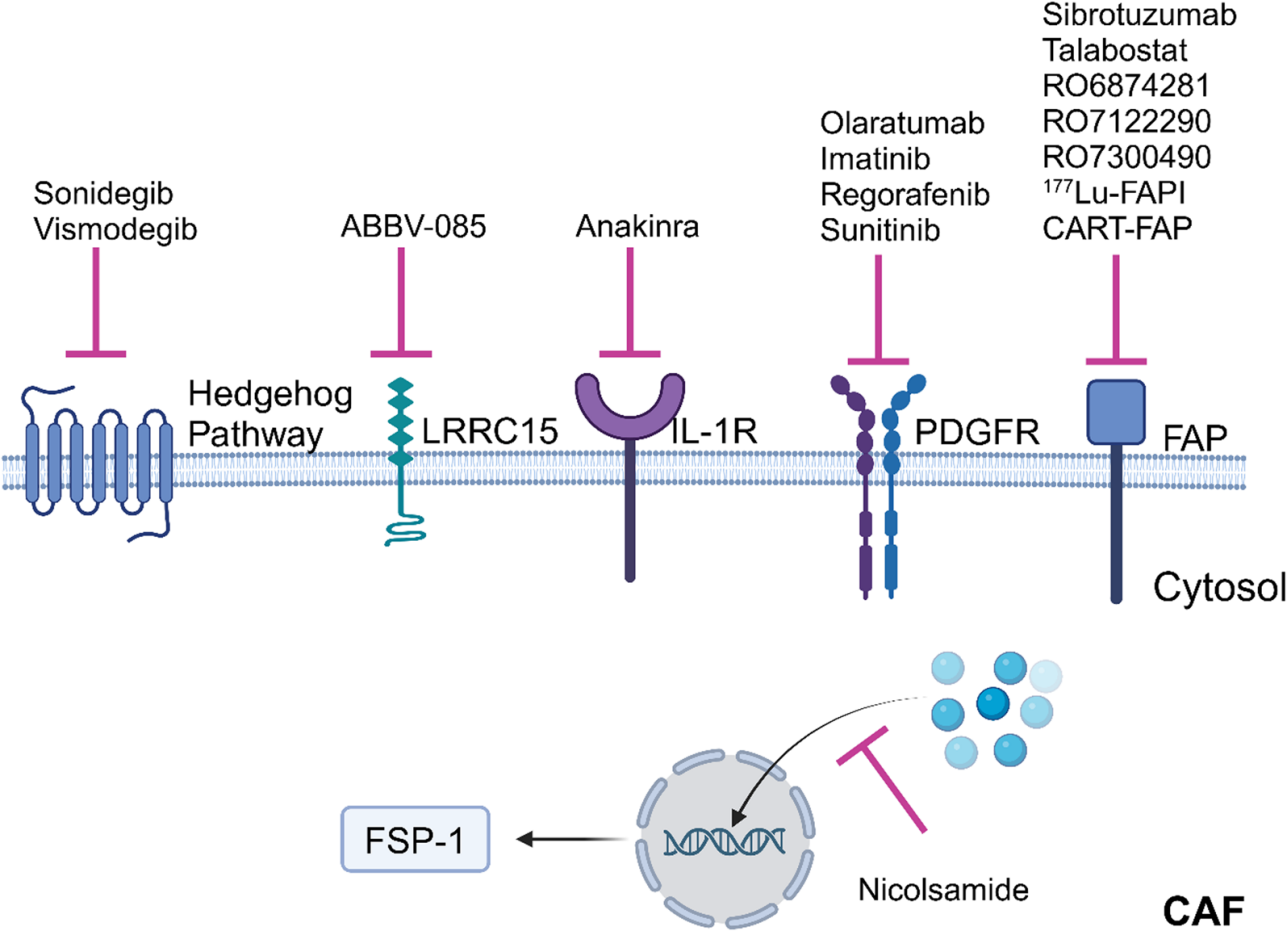
Treatments directly targeting CAFs currently in clinical trials. CAF, cancer-associated fibroblasts. Cell surface proteins and intracellular proteins highly expressed by CAFs have been targeted by various drugs. The figure was generated using BioRender

Given the pivotal roles of CAFs in cancer, various strategies have been proposed to develop therapeutic interventions targeting CAFs. These approaches primarily involve CAF elimination, reprogramming, and targeting functional factors originating from CAFs. It is essential to note that many treatments targeting CAFs do not exhibit direct inhibitory effects on cancer cells. Consequently, CAF-targeted therapies are often combined with other approaches against tumours, aiming to synergistically enhance their therapeutic efficacy. Proteins that are highly expressed by CAFs and play tumour-promoting roles are considered as attractive therapeutic targets. A common treatment strategy is to inhibit functions of these targets by using small molecular inhibitors or blocking antibodies (Fig. 3; Table 3).

**Table 3:** Clinical trials for therapies directly targeting CAFs

Targets	Agents	Combination	Cancer	Phase	Trial number	Outcomes	Refs.
FAP	Sibrotuzumab	Mono	CRC	II	NCT02198274	Futility	[273]
	Talabostat	Mono	CRC	II	–	SD (21%)	[274]
	Talabostat	Chemo	Melanoma	II	NCT00083252	ORR (12.5%)	[275]
	Talabostat	ICI	CRPC	Ib/II	NCT03910660	–	[276]
	Talabostat	ICI	PDAC	II	NCT05558982	–	[277]
	^177^Lu-FAP-2286	Mono	Solid	I/II	NCT04939610	Safe	[284]
	^177^Lu-EB-FAPI	Mono	Solid	I	NCT05400967	–	–
	^177^Lu-DOTA-FAPI	Mono	Solid	I	NCT04849247	Safe	[288]
	^177^Lu-DOTA-EB-FAPI	Mono	Thyroid	I	NCT05410821	DCR (83%) ORR (25%)	[289]
	^177^Lu-DOTA-EB-FAPI	Mono	Solid	I	NCT05963386	–	–
	^177^Lu-PNT6555	Mono	Solid	I	NCT05432193	–	–
	^177^Lu-LCN1004	Mono	Solid	I	NCT05723640	–	–
	CART-FAP	Mono	MPM	I	NCT01722149	Safe	[282]
	RO6874281	Mono	Solid	I	NCT02627274	Safe	[278]
	RO6874813	Mono	Solid	I	NCT02558140	Safe	[280]
	RO7122290	ICI	Solid	Ib/II	NCT04826003	ORR (18.4%)	[281]
	RO7300490	Mono, ICI	Solid	I	NCT04857138	–	–
PDGFR	Imatinib	ICI	Solid	I	NCT01738139	Safe	[293]
	Imatinib	ICI	Melanoma	Ib/II	NCT04546074	–	[294]
	Regorafenib	ICI	CRC	–	NCT04771715	SD (45%) PR (5%)	[321]
	Sunitinib	ICI	Sarcoma	1b/II	NCT03277924	PFS (48%)	[322]
	Olaratumab	Chemo	Sarcoma	III	NCT02451943	Futility	[296]
	Olaratumab	ICI	Sarcoma		NCT03126591	Safe	[297]
HH	Sonidegib	Mono	BCC	II	NCT01327053	ORR (48.1%)	[304]
	Sonidegib	Chemo	TNBC	I	NCT02027376	ORR (30%)	[305]
	Sonidegib	ICI	NSCLC	I	NCT04007744	Safe	[306]
	Vismodegib	Mono	BCC	II	NCT02667574	ORR (71%)	[307]
	Vismodegib	Mono	GC	II	NCT03052478	DCR (5.3%)	[308]
	Vismodegib	Chemo	Pancreatic	II	NCT01088815	Futility	[309]
FSP-1	Niclosamide	Mono	CRC	II	NCT02519582	Safe	[312]
LRRC15	ABBV-085	Mono	Solid	I	NCT02565758	ORR (20%)	[317]
IL-1R	Anakinra	Chemo, targeted	CRC	II	NCT02090101	SD (68.8%) ORR (15.6%)	[319]
	Anakinra	CAR-T	MM	1b/II	NCT03430011	Safe	[320]

CRC: colorectal cancer; CRPC: castration-resistant prostate cancer; PDAC: pancreatic ductal adenocarcinoma; MPM: malignant pleural mesothelioma; BCC: basal cell carcinoma; TNBC: triple negative breast cancer; NSCLC: non-small cell lung cancer; GC: gastric cancer; MM: multiple myeloma. SD: stable disease; ORR: objective/overall response rate; PR: partial response; PFS: progression free survival; DCR: disease control rate. Mono: monotherapy; Chemo: chemotherapy; Targeted: targeted therapy; ICI: immune checkpoint inhibitor

### FAP

Besides serving as a marker for CAFs, FAP is one of the most promising targets on CAFs, owing to its important roles and high expression in both CAFs and epithelial cells. Therapeutic treatments targeting FAP^+^ CAFs have shown capability to alleviate immunosuppression and enhance responses to ICIs. For instance, an adenoviral-vector vaccine designed to eliminate FAP^+^ cells reduced the number and suppressive function of immunosuppressive cells within tumours, concurrently inducing a robust CD8^+^ T cell response [263]. Talabostat, a small molecule dipeptidyl peptidase inhibitor of FAP, exhibited anti-tumour activity primarily through induction of tumour-specific cytotoxic T lymphocytes [264]. The introduction of CAR-T cells designed to target FAP also showed promising therapeutic outcomes in murine models [243, 265–267]. In addition, several studies explored targeted delivery of radioisotopes or drugs to tumours by using anti-FAP antibodies, resulting in therapeutic regressions in preclinical cancer models [268–270].

A number of treatments targeting FAP^+^ CAFs have progressed into clinical trials. The humanized murine anti-FAP monoclonal antibody F19 [271], known as Sibrotuzumab, showed a specific FAP targeting effect in cancer patients [272]. However, this antibody treatment alone did not demonstrate substantial therapeutic benefit in patients with metastatic CRC [273]. The FAP inhibitor talabostat also did not achieve significant therapeutic effect in the clinic either as a monotherapy or in combination with chemotherapy [274, 275]. Currently, an ongoing clinical investigation is exploring the combination of talabostat with immunotherapy [276, 277]. An anti-FAP bispecific antibody linked to IL-2v (RO6874281) has been assessed in a Phase I trial, showing objective responses in some patients [278]. Another bispecific antibody, targeting both FAP and DR5, displayed strong anti-tumour efficacy in preclinical models [279] and is currently under clinical evaluation [280]. Other anti-FAP bispecific antibodies featured with FAP-targeting and immunomodulatory effects have also been developed [281]. The exploration of CAR-T cell therapies target FAP is underway in clinical trials too [282].

Recent developments in targeted delivery of radionuclide to tumours by anti-FAP peptides or inhibitors have shown great promise [283, 284]. Several peptide-based FAP inhibitors (FAPI) with high affinities and selective binding to FAP-expressing tumours have been developed. Radiolabelled FAPI with ^177^Lu exhibited promising efficacy in preclinical cancer models [285–287], leading to the assessment of ^177^Lu-FAPI in clinical trials. Safety profiles of ^177^Lu-FAPI have been established in several phase I studies, and anti-tumour effects were observed in cancer patients [284, 288]. In a dose-escalation study for treating patients with metastatic radioiodine refractory thyroid cancer, ^177^Lu-FAPI demonstrated promising therapeutic efficacy, with a disease control rate (DCR) of 83% and objective response rate (ORR) of 25% [289]. More ongoing clinical trials are in progress to evaluate the efficacy of different ^177^Lu-FAPI treatments. In the future, it will be interesting to explore the therapeutic effect of combining ^177^Lu-FAPI with established anti-tumour therapies like immunotherapy and chemotherapy.

### PDGFRα/β

High expression of PDGFRα/β has been observed in CAFs, vascular cells, and malignant cells, where these receptors play crucial roles in shaping an immunosuppressive TME, promoting angiogenesis, facilitating tumour growth, and fostering metastasis [290]. Therapeutic interventions targeting PDGFRα/β demonstrated potential to enhance immunotherapies in murine models, prompting further exploration in clinical settings [291, 292].

Several small molecule inhibitors targeting the PDGF/PDGFR pathway have been developed. Imatinib, a TKI targeting PDGFR, c-Kit, and BCR-ABL, has been approved by the U.S. Food and Drug Administration (FDA) for treating several cancers. Clinical trials are currently exploring the combined use of imatinib with immunotherapies. While a combination of imatinib and ipilimumab was well-tolerated in cancer patients, it did not exhibit a synergistic effect [293]. Efficacy studies involving the combination of imatinib with other ICIs, such as atezolizumab and pembrolizumab, are currently underway [294]. Other multi-target TKIs for PDGFR, including regorafenib, sunitinib, ripretinib, and avapritinib, have received FDA approval for treating gastrointestinal stromal tumours (GIST) [295]. These TKIs are also being evaluated as combination treatments with immunotherapies or targeted therapies in different cancers.

In addition to small molecule inhibitors, there is ongoing development of antibodies targeting PDGFR. The combination of the anti-PDGFRα antibody olaratumab with doxorubicin did not yield a significant improvement in OS for sarcoma patients compared to the placebo plus doxorubicin treatment [296]. The combination of olaratumab with pembrolizumab was well-tolerated, with a reported disease control rate (DCR) of 53.6% in a phase I trial [297]. Bispecific antibodies concurrently binding to PDGFR and other targets also showed promising results in preclinical studies [298, 299], while their clinical effectiveness remain to be investigated.

### Hedgehog (HH) signalling

Activation of the HH signalling pathway in CAFs promoted tumorigenesis and metastasis in preclinical studies [300, 301]. In contrast, inhibition of the HH signalling pathway using the specific inhibitor sonidegib reduced the myCAF/iCAF ratio and impeded tumour growth [302]. Another HH inhibitor, vismodegib, also exhibited inhibitory effects on tumour growth in preclinical models [303]. These promising findings led to the evaluation of HH inhibitors in clinical trials. Sonidegib demonstrated robust efficacy in a phase II trial involving patients with basal cell carcinoma [304]. The combination of sonidegib and chemotherapy showed anti-tumour activity in triple-negative breast cancer (TNBC) patients [305]. Currently, the efficacy of combining sonidegib with pembrolizumab is under investigation for treating NSCLC [306]. Vismodegib, as a monotherapy, achieved a 71% ORR in basal cell carcinoma patients [307] and a DCR of 5.3% in gastric cancer patients [308]. In newly diagnosed metastatic pancreatic cancer patients, the combination of vismodegib and chemotherapy did not enhance efficacy of chemotherapy [309]. To facilitate future clinical trials for therapies targeting HH signalling, it will be beneficial to develop diagnostic approaches to evaluate HH signalling activation in cancer patients.

### Other CAF targets

Other proteins highly expressed by CAFs have also been targeted in preclinical studies and clinical trials. For instance, niclosamide functioning as a FSP-1 transcriptional inhibitor, demonstrated potential in reducing liver metastasis of colon cancer and boosting efficacy of ICIs in preclinical models [310, 311]. Niclosamide exhibited favourable tolerability in patients and is being evaluated in a phase II trial for CRC [312]. Neutralising antibodies targeting PDPN showed inhibition of tumour growth and metastasis in xenograft models for osteosarcoma, oral cancer, and malignant pleural mesothelioma (MPM) [313–315], supporting clinical assessment of anti-PDPN antibodies in the future. The tumour-promoting characteristic of CAFs with high LRRC15 expression has inspired the development of LRRC15-targeted therapies. ABBV-085, a monomethyl auristatin-E (MMAE) antibody–drug conjugate targeting LRRC15, demonstrated anti-tumour efficacy in preclinical models [316]. The safety and tolerability of ABBV-085 in patients were assessed, with reported anti-tumour responses in sarcoma patients [317]. CAFs with elevated IL-1R expression were shown to promote tumour development and induce an immunosuppressive TME [241]. Anakinra, an FDA-approved IL-1R antagonist for treating rheumatoid arthritis, showed potential to reduce CAF-derived thymic stromal lymphopoietin, which correlates with poor survival rates in pancreatic cancer patients [318]. Encouragingly, combination of anakinra with 5-FU and bevacizumab has shown promise in treating patients with refractory CRC [319]. Currently, a phase Ib/II clinical trial is underway to explore the combination of anakinra with CAR-T therapy for the management of relapsed multiple myeloma [320]. As many of these targets may not be widely expressed in all cancer patients, a stringent selection of cancer patients for clinical trials is essential for future studies.

## Targeting CAF-derived factors

**Fig. 4:**
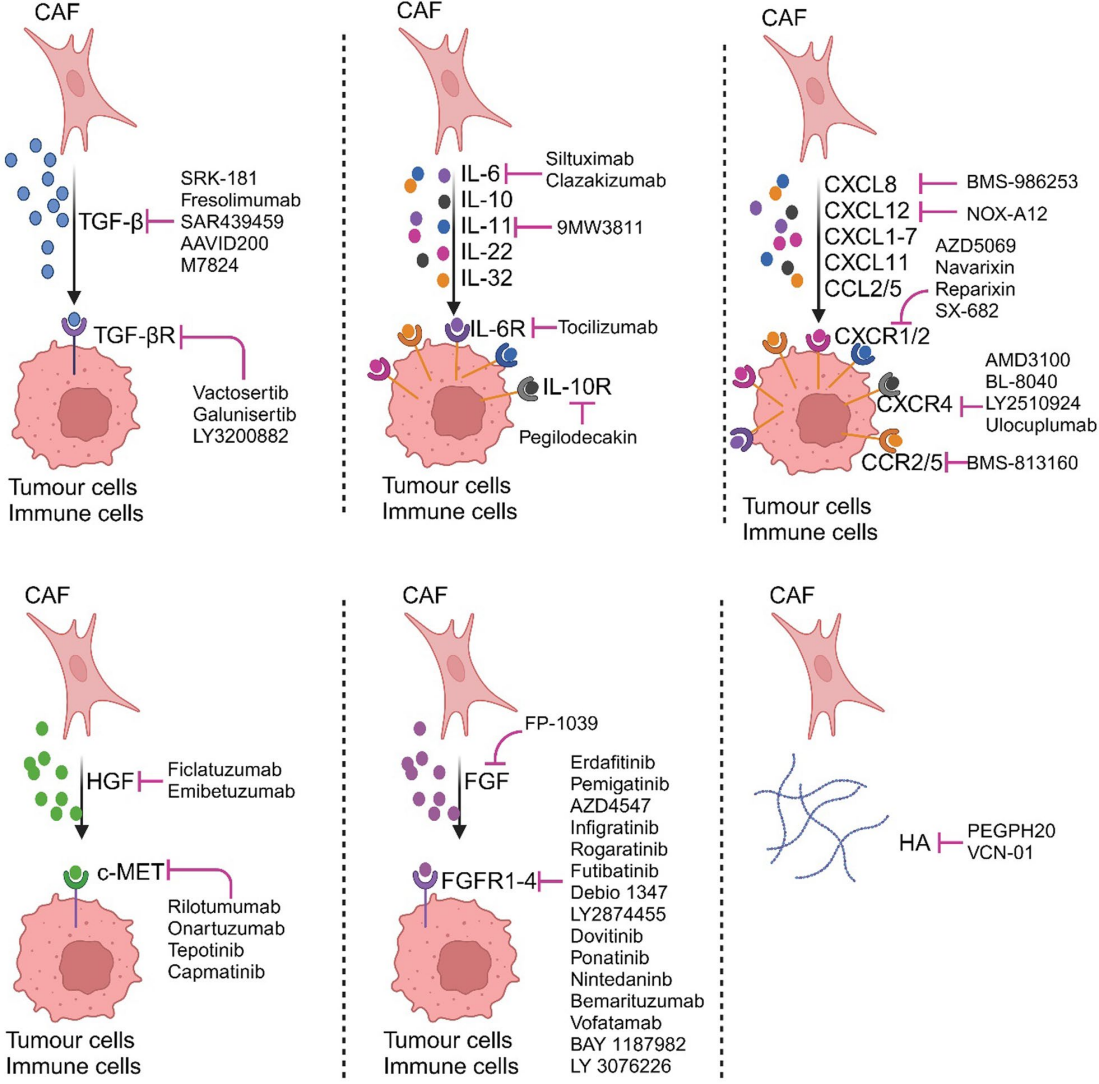
Drugs targeting CAF-derived factors that promote tumour development. CAF: cancer-associated fibroblasts. The figure was generated using BioRender

Many tumour-promoting factors derived from CAFs have been identified in the past, positioning them as promising targets for therapeutic interventions. These factors can either directly interact with cancer cells to regulate tumour actions, or affect other stromal components like immune cells. Many clinical trials have been carried out to assess the efficacy of drugs targeting these CAF-derived factors (Fig. 4; Table 4).

**Table 4:** Clinical trials for therapies targeting CAF-derived factors and corresponding receptors

Targets	Agents	Comb	Cancer	Phase	Trial number	Outcomes	Refs.
TGF-β	SRK-181	Mono; ICI	Solid	I	NCT04291079	Safe	[325]
	Fresolimumab	Mono	Melanoma; RCC	I	NCT00356460	Safe	[326]
	Fresolimumab	Mono	Glioma	I	NCT01472731	-	[328]
	Fresolimumab	Mono	MPM	II	NCT01112293	SD (23.1%)	[327]
	SAR439459	Mono; ICI	Solid	I	NCT03192345	Bleeding risk	[330]
	AVID200	Mono	Solid	I	NCT03834662	Safe	[332]
	M7824	Mono	NSCLC	III	NCT03631706	No improved efficacy	[335]
TGF-βR	Vactosertib	Mono	Solid	I	NCT02160106	Safe	[337]
	Vactosertib	Targeted	Desmoid	Ib/II	NCT03802084	Safe	[338]
	Vactosertib	Targeted	MM	I	NCT03143985	Safe	[342]
	Galunisertib	Targeted	HCC	II	NCT01246986	Prolonged OS	[339]
	Galunisertib	Chemo	Pancreatic	Ib/II	NCT01373164	Prolonged OS	[340]
	LY3200882	Mono; ICI; Chemo; Radio	Solid	I	NCT02937272	Safe	[341]
IL-6	Siltuximab	Mono	Prostate	II	NCT00433446	SD (23%)	[348]
	Clazakizumab	Mono	NSCLC	II	NCT00866970	–	[349]
	Siltuximab	ICI	Pancreatic	Ib/II	NCT04191421	–	[352]
	Siltuximab	Chemo	MM	Ib/II	NCT01531998	ORR (90.9%) CR (9.1%) PR (81.8%)	[353]
IL-6R	Tocilizumab	ICI	Lung	Ib/II	NCT04691817	–	–
	Tocilizumab	ICI	Melanoma	II	NCT03999749	–	–
IL-10R	Pegilodecakin	ICI	NSLCL	Ib	NCT02009449	ORR (43%)	[359]
	Pegilodecakin	Chemo	PDAC	III	NCT02923921	Futility	[360]
IL-11	9MW3811	Mono	Solid	I	NCT05911984	–	–
CXCL8	BMS-986253	ICI	Solid	Ib/II	NCT03400332	PR (17.9%)	[365]
CXCL12	NOX-A12	Mono; ICI	CRC; Pancreatic	Ib/II	NCT03168139	SD (25%)	[373]
	NOX-A12	Radio	GBM	Ib/II	NCT04121455	PR (40%)	[374]
CCR2/5	BMS-813160	ICI	PDAC	Ib/II	NCT03767582	Safe	[378]
CXCR2	AZD5069	Hormone	CRPC	Ib/II	NCT03177187	PR (24%)	[381]
	Navarixin	ICI	Solid	II	NCT03473925	–	–
CXCR1/2	Reparixin	Mono	TNBC	II	NCT01861054	Futility	[384]
	SX-682	ICI	Solid	Ib/II	NCT04574583	Bleeding risk	[385]
	SX-682	ICI	Pancreatic	I	NCT04477343	–	[386]
	SX-682	ICI	CRC	Ib/II	NCT04599140	–	[387]
CXCR4	AMD3100	Targeted	MM	Ib/II	NCT00903968	ORR (48.5%)	[389]
	BL-8040	Chemo; ICI	Pancreatic	II	NCT02826486	ORR (32%) DCR (77%)	[390]
	LY2510924	Targeted	RCC	II	NCT01391130	Futility	[391]
	LY2510924	Chemo	SCLC	II	NCT01439568	Futility	[392]
	Ulocuplumab	Chemo; Targeted	MM	Ib/II	NCT02666209	ORR (55.2%)	[393]
CXCL9/10	NG-641	Mono	Solid	I	NCT04053283	Safe	[397]
HGF	Ficlatuzumab	Targeted	HNSCC	II	NCT03422536	ORR (19%)	[399]
	Ficlatuzumab	Targeted	Lung	Ib/II	NCT01039948	Futility	[400]
	Emibetuzumab	Mono	NSCLC	II	NCT01900652	ORR (4.3%)	[401]
	Emibetuzumab	Targeted	Solid	Ib/II	NCT02082210	DCR (60%) ORR (6.7%)	[402]
c-MET	Rilotumumab	Chemo	Gastric	III	NCT01697072	Worse outcome	[403]
	Onartuzumab	Targeted	NSCLC	II	NCT00854308	Improved OS and PFS	[404]
	Tepotinib	Mono	HCC	Ib/II	NCT01988493	ORR (10.5%)	[406]
	Capmatinib	ICI	NSCLC	II	NCT04139317	Futility	[407]
FGF	FP-1039	Chemo	MPM	Ib	NCT01868022	ORR (36%) SD (47%) DCR (86%) PR (14/36)	[410]
	FP-1039	Chemo	NSCLC	Ib	NCT01868022	ORR (47%)	[411]
FGFR	Pemigatinib	Mono	CCA	II	NCT02924376	ORR (35.5%) DCR (82%)	[415]
	AZD4547	Mono	Breast	Ib/II	NCT01791985	ORR (10%)	[416]
	AZD4547	Mono	Solid	II	NCT02465060	ORR (5%) SD (51%)	[417]
	Infigratinib	Mono	CCA	II	NCT02150967	ORR (23.1%)	[418]
	Infigratinib	Mono	GBM	II	NCT01975701	ORR (3.8%)	[419]
	Debio 1347	Mono	Solid	I	NCT01948297	ORR (16.7%) DCR (79%)	[422]
	Dovitinib	Mono	RCC	I	NCT00715182	ORR (3.0%) DCR (49.3%)	[424]
	Nintedanib	Chemo	NSCLC	III	NCT00805194	ORR (4.4%) DCR (54.0%)	[426]
	Rogaratinib	Mono	UCC	IIb/III	NCT03410693	ORR (20.7%)	[420]
	Futibatinib	Mono	Solid	Ib/II	NCT04189445	ORR (11.5%)	[421]
	LY2874455	Mono	Solid	I	NCT01212107	DCR (85.2%)	[423]
	Erdafitinib	Mono	UCC	II	NCT02365597	ORR (40%)	[413]
	Erdafitinib	Mono	CCA	II	NCT02699606	ORR (40.9%) DCR (81.8%)	[414]
	Ponatinib	Mono	GIST	II	NCT01874665	ORR (7%)	[425]
	Bemarituzumab	Chemo	Gastric	II	NCT03694522	ORR (53%)	[428]
	Vofatamab	ICI	UCC	Ib/II	NCT03123055	ORR (29.6%)	[429]
	BAY 1187982	Mono	Solid	I	NCT02368951	Poor tolerability	[430]
	LY3076226	Mono	Solid	I	NCT02529553	Safety dose	[431]
HA	PEGPH20	Chemo	PDAC	Ib/II	NCT01959139	Reduced OS	[436]
	PEGPH20	Chemo	PDAC	III	NCT02715804	ORR (47%) No effect on OS and PFS	[437]
	PEGPH20	ICI	PDAC	II	NCT03634332	Increased medium OS	[438]
	PEGPH20	ICI	PDAC	Ib/II	NCT03193190	ORR (6.1%)	[439]
	PEGPH20	ICI	GC	Ib/II	NCT03281369	Futility	[439]
	VCN-01	Chemo	PDAC	I	NCT02045602	ORR (50%)	[440]

RCC: Renal Cell Carcinoma; MPM: Malignant Pleural Mesothelioma; NSCLC: non-small cell lung cancer; MM: multiple myeloma; HCC: hepatocellular carcinoma; PDAC: pancreatic ductal adenocarcinoma; GBM: glioblastoma; CRC: colorectal cancer; CRPC: castration-resistant prostate cancer; TNBC: triple negative breast cancer; SCLC: Small Cell Lung Cancer; HNSCC: head and neck squamous cell carcinoma; UCC: urothelial carcinoma; CCA: cholangiocarcinoma; GIST: gastrointestinal stromal tumour; GC: gastric cancer. OS: overall survival; SD: stable disease; ORR: objective/overall response rate; CR: complete response; PR: partial response; PFS: progression free survival; DCR: disease control rate. Radio: radiotherapy

### TGF-β

CAF-mediated TGF-β signalling pathway is involved in the crosstalk between CAFs and cancer cells. Activation of the TGF-β signalling pathway in cancer cells can increase proliferation, migration, invasion, immunosuppression, and therapy resistance. By inhibiting activation of latent TGF-β1, the agent SRK-181-mIgG1 can sensitise tumour response to anti-PD-1 treatment in preclinical models, without causing evident toxicities [323, 324]. In a phase I study, SRK-181 exhibited no dose-limiting toxicity when administered as a monotherapy or in combination with pembrolizumab [325], while the efficacy remains to be explored. Fresolimumab, a neutralizing monoclonal antibody for all TGF-β isoforms, exhibited good tolerance and anti-tumour activity in a phase I trial [326]. However, its immunoregulatory effects were found to be minimal in a subsequent phase II study [327]. An imaging study utilizing ^89^Zr radiolabelled fresolimumab demonstrated good penetration into recurrent high-grade gliomas, but the antibody did not yield clinical benefits, leading to discontinuation of further development for oncology indications [328]. Another anti-TGF-β monoclonal antibody SAR439459 demonstrated a synergistic effect with PD-1 blockade, enhancing anti-tumour immunity in a preclinical study [329]. Unfortunately, a recent study revealed a lack of efficacy and a notable risk of bleeding in cancer patients treated with this drug, resulting in termination of the trial [330].

An alternative strategy for targeting TGF-β involves designing ligand traps. AVID200, a receptor ectodomain trap computationally designed to target TGF-β1/3, increased T-cell-mediated cytotoxicity and enhanced the efficacy of ICIs in syngeneic preclinical models [331]. The safety prolife of AVID200 is currently under clinical evaluation [332]. Bifunctional molecules containing TGF-β traps have also been developed, and one notable example is M7824 that combines the TGF-βRII receptor (acting as a trap) with an anti-PD-L1 IgG1 [333]. Preclinical studies have demonstrated the tumour-targeting effect and anti-tumour efficacy of M7824 [333, 334]. However, a phase III clinical trial was terminated due to a lack of superior efficacy compared to pembrolizumab [335]. Another bifunctional TGF-β trap fused drug, anti-CTLA4-TGF-βRII, showed superior anti-tumour efficacy compared to an anti-CTLA4 antibody alone in preclinical models [336], but its efficacy in patients has not been investigated.

The cytoplasmic kinase activity of TGF-β receptors can also be targeted for cancer therapy. Several small molecule receptor kinase inhibitors have been developed for this purpose and are currently in clinical trials [337–342]. For example, vactosertib, an orally bioavailable TGF-β receptor kinase inhibitor, showed efficacy against multiple myeloma in preclinical models, either as a monotherapy or in combination with other treatments [343, 344], leading to the clinical assessment of vactosertib. Similar drugs such as galunisertib and LY3200882 are under clinical investigation.

### IL-6

CAF-derived IL-6 contributes to cancer invasion, metastasis, angiogenesis, immune modulation, and drug resistance. Several drugs targeting IL-6 or the IL-6 receptor (IL-6R) received FDA approval for treating inflammatory diseases like rheumatoid arthritis [345]. Recently, their potential in cancer therapy has attracted attention, with observed anti-tumour efficacy in preclinical models [346, 347]. One such drug, siltuximab, a chimeric anti-IL-6 antagonistic antibody, received FDA approval for treating multicentric Castleman disease and is currently being investigated for treating cancers. In patients with castration-resistant prostate cancer (CRPC), elevated baseline IL-6 was correlated with poor survival, and siltuximab treatment resulted in a 23% stable disease (SD) rate [348]. Another anti-IL-6 antibody, clazakizumab, improved cancer cachexia in NSCLC patients, as shown by biomarker analysis [349]. In preclinical models resistant to anti-PD-L1 treatment, dual blockade of IL-6R and PD-L1 attenuated tumour growth and improved survival [350, 351], leading to clinical evaluation of this combination therapy. A combination of siltuximab and spartalizumab is currently in a phase Ib/II trial for metastatic pancreatic cancer [352]. Combination of siltuximab with chemotherapies achieved an impressive ORR of 90.9% in patients with untreated multiple myeloma [353]. Tocilizumab, an anti-IL-6R humanized monoclonal antibody, is also under clinical investigation in combination with ICIs.

### Other interleukins

In addition to IL-6, CAFs can produce many other interleukins, including IL-10, IL-11, IL-22, IL-32, and inhibiting actions of these interleukins resulted in anti-tumour effects in some studies [22, 354–356]. For instance, neutralising IL-10 with an antibody potentiated anti-tumour immune reaction in a preclinical model mimicking human CRC liver metastases [357]. Interestingly, overexpression of IL-10 or administration of pegylated IL-10 in preclinical models also inhibited tumour growth [358]. Pegilodecakin, acting as an IL-10 receptor agonist, exhibited a notable 43% ORR in NSCLC patients when combined with nivolumab or pembrolizumab [359]. However, in another clinical study, addition of pegilodecakin failed to improve the efficacy of chemotherapy in advanced PDAC patients [360]. Therapeutics targeting IL-11/IL-11R signalling are recently developed, with a humanised anti-IL-11 antibody 9MW3811 currently in a phase I trial for treating solid tumours. Treatments targeting IL-22/IL-22R or IL-32/IL-32R signalling have not yet been developed.

### CXC chemokines

CAFs secrete a range of C-X-C motif chemokine ligand (CXCL) family proteins that act on cancer cells and stromal cells, leading to increased tumour proliferation, metastasis, and immunosuppression. Preclinical studies have demonstrated great potential in targeting CXCL chemokines for cancer therapy. Inhibiting CAF-derived CXCL1 using antagonistic antibodies reversed radio-resistance in oesophageal squamous cell carcinoma xenograft models [361] and reduced growth of bladder cancer cells [362]. Another humanised monoclonal antibody NTC-001 neutralising CXCL1, is currently undergoing preclinical evaluation [363]. CAF-derived CXCL8 (also known as IL-8) can promote tumour resistance to cisplatin in gastric cancer [364]. An anti-CXCL8 neutralizing antibody BMS-986253, when combined with nivolumab, showed tolerable safety and resulted in partial response (PR) in cancer patients who had previously progressed after anti-PD-(L)1 or anti-CTLA-4 treatment [365]. The role of CXCL11 in tumour development is controversial. CAF-derived CXCL11 increased migration and metastasis of HCC [366], while cancer cell-secreted CXCL11 enhanced CD8^+^ T cell infiltration in a preclinical study [367]. Elevated levels of CXCL11 were associated with anti-tumour immune responses and improved prognosis in colon cancer [368]. CXCL12 secreted by CAFs contributes to tumour proliferation, invasion, metastasis, immunosuppression, and angiogenesis [369–372]. Combining a CXCL12 inhibitor, NOX-A12, with pembrolizumab induced immune response, resulting in SD in heavily pretreated cancer patients [373]. In addition, combining NOX-A12 with radiotherapy led to partial remission of target lesions in GBM patient [374]. While roles for CXCL2, CXCL3, CXCL5, and CXCL7 in cancers have been reported [46, 375, 376], specific treatments targeting these chemokines have not yet been developed. CAFs also produce CCL2 and CCL5, two other chemokine ligands promoting tumour growth and metastasis [49, 377]. BMS-813160, a dual antagonist targeting CCR2 and CCR5 (the receptors for CCL2 and CCL5), is currently under assessment for efficacy in combination with nivolumab [378].

Some treatments have been developed to target receptors of CXC ligands, considering the capacity of CXC receptors (CXCRs) in binding to multiple CXC ligands. For instance, CXCR2 is known to interact with seven CXCL proteins, including CXCL1, CXCL2, CXCL3, CXCL5, CXCL6, CXCL7, and CXCL8 [379]. Several antagonists targeting CXCR2 are currently under clinical evaluation. AZD5069, a CXCR2 inhibitor, exhibited promising anti-tumour activity in patients with metastatic CRPC when combined with enzalutamide [380, 381]. Ongoing investigations are exploring the efficacy of AZD5069 in combination with ICIs. Additionally, some CXCR2 inhibitors, such as danirixin and elubrixin, which were initially developed for treating non-cancer diseases, are being repurposed for cancer treatment with encouraging prospects. CXCR1 as a receptor for CXCL6 and CXCL8, is also a promising target for cancer treatment. Reparixin, which was initially developed as a CXCR1/2 inhibitor to attenuate inflammatory responses in organ transplantation and tissue injury [382], demonstrated anti-tumour effects in preclinical models [383]. Unfortunately, a clinical trial assessing the efficacy of reparixin in treating TNBC was terminated due to lack of efficacy [384]. SX-682, another CXCR1/2 inhibitor, when combined with M7824 and CV301 (a vaccine for CEA and MUC1), resulted in disease controls in some patients but also caused grade 3 bleeding adverse effect [385]. Combination of SX-682 with other ICIs is currently assessed in phase I/II trials [386, 387]. CXCR4, the receptor for CXCL12, is also being targeted for cancer therapy in the clinic. AMD3100 as a CXCR4 antagonist was approved by FDA for autologous transplantation in patients with non-Hodgkin’s Lymphoma or multiple myeloma [388]. The combination of AMD3100 with bortezomib resulted in a clinical benefit rate of 60.6% and an ORR of 48.5% in pretreated multiple myeloma patients [389]. BL-8040, a cyclic peptide inhibitor for CXCR4, when combined with pembrolizumab and chemotherapy, demonstrated a DCR of 77% in pancreatic cancer patients [390]. However, another cyclic peptide inhibitor for CXCR4, LY2510924, did not improve the efficacy of sunitinib in patients with RCC [391], and was ineffective in SCLC patients [392]. Notably, an anti-CXCR4 antagonist antibody, ulocuplumab, resulted in a 55.2% ORR and a clinical benefit rate of 72.4% when combined with lenalidomide and dexamethasone [393].

In contrast to the tumour promoting CXCL proteins, some CXC chemokines exhibit anti-tumour activity. These chemokines are usually secreted by cancer cells or other stromal cells rather than CAFs. Notably, CXCL9 and CXCL10 inhibited tumour growth and enhanced the efficacy of ICIs in preclinical cancer models [394–396]. These findings has led to the development of NG-641, an oncolytic adenoviral vector engineered to encode four immunostimulatory transgenes, including CXCL9, CXCL10, IFNα, and a bispecific T cell activator antibody targeting both FAP and CD3 [397]. The safety profile of NG-641 is currently under phase I clinical assessment, with no result released at the current stage.

### HGF

HGF produced by CAFs can activate the c-MET receptor tyrosine kinase on tumour cells, promoting tumour growth and metastasis [398]. The humanised anti-HGF antagonistic antibody ficlatuzumab did not yield clinical benefits as a monotherapy, but resulted in a 19% ORR in patients with HNSCC when combined with cetuximab [399]. In another study, combining ficlatuzumab with gefitinib showed no significant difference compared to gefitinib monotherapy [400]. Emibetuzumab, another anti-HGF antagonistic antibody, was well tolerated but achieved only 4.3% ORR in patients with MET-positive NSCLC [401]. Combining emibetuzumab with ramucirumab (an anti-VEGFR2 antibody) resulted in a 6.7% ORR and a 60% DCR in HCC patients [402].

The c-MET receptor tyrosine kinase was also targeted for treating different cancers. Unfortunately, rilotumumab, a c-MET targeting agent, failed to meet the primary endpoint and was associated with worse OS in a phase III study [403]. However, the combination of erlotinib and onartuzumab, another antagonistic antibody for c-MET, resulted in improvements in both progression-free survival and OS in MET-positive NSCLC patients [404]. The FDA has now approved capmatinib and tepotinib (two highly selective MET inhibitors) for treating metastatic NSCLC with MET exon 14 skipping [405]. Tepotinib monotherapy was also more effective than sorafenib (targeting VFGFR, PDGFR, c-Kit, RET) in treating HCC patients with MET-positive tumours [406]. The combination therapy of capmatinib and pembrolizumab was not well tolerated and did not enhance ICI efficacy in NSCLC patients [407]. These studies suggest that treatments targeting HGF-c-Met signalling may only be effective to a fraction of cancer patients that need to be carefully selected in future clinical trials.

### FGF

FGF proteins secreted by tumour stromal cells interact with FGF receptors (FGFRs) on cancer cells, resulting in enhanced cancer cell growth [408]. Aberrant activation of FGFR in cancer has been observed and can occur through variants, gene fusion, and copy number amplification [409]. Considering the important roles of FGF/FGFR signalling in cancer, treatments targeting this signalling have been developed. An example is FP-1039, which serves as a FGF ligand trap consisting of a Fc region and extracellular domain of FGFR1. FP-1039 treatment showed a 36% ORR in MPM and a 47% ORR in NSCLC in a phase Ib study [410, 411]. Due to the versatility of FGFR in binding different FGFs, interventions have also been developed to inhibit actions of FGFR. Erdafitinib and pemigatinib, two TKIs targeting FGFR1-4 and FGFR1-3 respectively, obtained FDA approval for treating advanced urothelial cancer with FGFR2/3 genetic alterations and myeloid/lymphoid neoplasms with FGFR1 rearrangement [412]. In a phase II trial, erdafitinib demonstrated a 40% ORR in patients with advanced or metastatic urothelial cancer harbouring FGFR alterations [413]. Comparable results were reported in cholangiocarcinoma (CCA) patients with FGFR alterations, in which erdafitinib achieved a 40.9% ORR in a phase IIa study [414]. Pemigatinib, in comparation, resulted in a 35.5% ORR in CCA patients with FGFR2 fusions or rearrangements [415].

AZD4547, a selective inhibitor of FGFR1-3, showed a 10% ORR in patients with endocrine-resistant breast cancer [416], and a 5% ORR in solid tumours with aberrations in FGFR pathway [417]. Other selective inhibitors for FGFR have also been evaluated in the clinic, exhibiting variable efficacy [418–423]. Non-selective inhibitors targeting FGFR have also been explored in the clinic. For instance, dovitinib targeting FGFR1/3, VEGFR1/3, c-KIT, FLT, showed a 3.0% ORR and a 49.3% DCR in advanced and metastatic RCC [424]. Ponatinib, which targets FGFR1 and other tyrosine kinases, exhibited a 7% ORR in GIST with KIT mutations after the failure of TKI treatment [425]. Nintedanib, an FDA-approved drug targeting FGFR1-3, VEGFR1-3, PDGFRα/β, FLT3, could enhance the efficacy of docetaxel in NSCLC [426]. More non-selective FGFR inhibitors have been reported and summarised by others [409, 427].

Antagonistic antibodies targets FGFR have also been developed and evaluated. Bemarituzumab targeting FGFR2b achieved a 53% ORR in gastric cancer harbouring FGFR2 overexpression or amplification [428]. Another antibody targeting FGFR3 showed a 29.6% ORR when combined with pembrolizumab for treating metastatic urothelial cancer [429]. Recent advancements on FGFR targeted therapy also include two antibody–drug conjugates, BAY1187982 and LY3076226. The BAY1187982 targeting FGFR2 to deliver auristatin-based payloads, showed poor tolerability in a phase I trial, leading to termination of this study [430]. In contrast, LY3076226 targeting FGFR3 with a cleavable linker and the maytansine derivative DM4 payload, exhibited acceptable safety and tolerability, but no responses were observed [431]. In the future, the combination of these drugs with other treatments could be explored.

### Hyaluronan (HA)

CAFs also produce high-molecular-mass polysaccharides like HA to regulate cancer behaviours [432, 433]. The HA forms substantial complexes with proteoglycans, contributing to increased tumour interstitial fluid pressure, which limits penetration of therapeutic treatments into tumours [434]. Enzymatic depletion of HA with a recombinant HA-degrading enzyme resulted in reduced tumour cell ECM, decreased interstitial fluid pressure, decompression of tumour vessels, increased tumour vascular area, inhibited tumour growth, and enhanced chemotherapy efficacy [435]. These findings promoted clinical investigation of a HA-degrading enzyme, PEGPH20, in combination with other anti-cancer therapies. Unfortunately, the combination of PEGPH20 with chemotherapy resulted in increased toxicity and decreased OS in general PDAC patients [436]. Another study involving PDAC patients with elevated HA levels showed that combining PEGPH20 with chemotherapy cannot improve OS and progression-free survival [437]. However, when combined with pembrolizumab, PEGPH20 improved OS in HA-high PDAC patients [438]. The combination of PEGPG20 with atezolizumab showed very limited activity in PDAC and no benefit in GC patients [439]. Interestingly, VCN-01, an oncolytic virus expressing hyaluronidase, showed encouraging clinical activity in PDAC, achieving an ORR of 50% in a phase I trial [440], implying that the delivery method for HA-degrading enzyme could make a difference in therapeutic outcomes. In contrast to the systemically delivery of PEGPH20, the VCN-01 has the unique capability to induce local tumour production of hyaluronidase, potentially resulting in a more targeted and effective distribution of the enzyme in tumours.

## CAF reprogramming

**Fig. 5:**
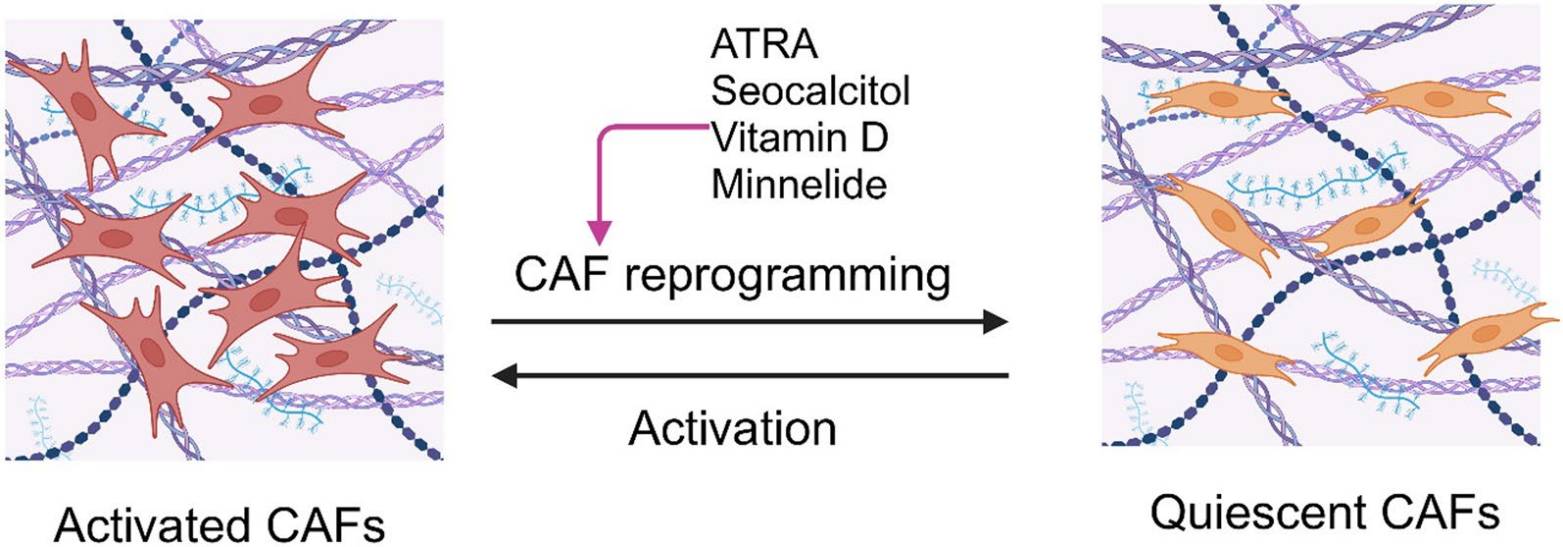
Drugs aiming to reprogram activated CAFs into quiescent CAFs. The figure was generated using BioRender

Reprogramming activated CAFs into quiescent CAFs is another strategy for cancer therapy targeting CAFs in TME (Fig. 5; Table 5). This approach could be promising for treating pancreatic cancer, where ablation of CAFs unexpectedly accelerated tumour growth in preclinical models. All-Trans Retinoic Acid (ATRA) as a standard treatment for patients with acute promyelocytic leukemia, could transform activated CAFs into quiescent CAFs. In pancreatic cancer, ATRA binds to retinoic acid receptor beta on pancreatic stellate cells, suppressing ECM remodelling and inhibiting tumour cell invasion [441]. Combining ATRA with gemcitabine led to enhanced anti-tumour effect in KPC mice [442]. The combination of ATRA with gemcitabine-nab-paclitaxel was safe and well tolerated in PDAC patients, resulting in a median OS longer than previously reported for chemotherapy-only treatments [443]. In addition, the combination of ATRA with pembrolizumab exhibited an ORR of 71% and a 50% complete response in patients with metastatic melanoma [444]. Additional studies are required to investigate whether the addition of ATRA can augment the efficacy of other therapies in different cancer types.

**Table 5:** Clinical trials for therapies aiming to deactivate CAFs

Agents	Comb	Cancer	Phase	Trial number	Outcomes	Refs.
ATRA	Chemo	PDAC	I	NCT03307148	Prolonged median OS	[443]
ATRA	ICI	Melanoma	Ib/II	NCT03200847	ORR (71%)	[444]
Seocalcitol	Mono	Pancreatic	II	–	Futility	[448]
Vitamin D	Chemo	CRC	II	NCT01516216	Futility	[449]
Minnelide	Mono	Pancreatic	II	NCT03117920	–	–
Minnelide	Mono	GIST	I	NCT01927965	–	–
Minnelide	Chemo	PDAC	I	NCT05557851	–	–

PDAC, pancreatic ductal adenocarcinoma; CRC, colorectal cancer; GIST, gastrointestinal stromal tumour. OS, overall survival; ORR, objective/overall response rate

Vitamin D treatment also showed potential to deactivate CAFs and reduce the production of tumour-promoting factors [445, 446]. In patient with early stage lung adenocarcinoma and low vitamin D level, vitamin D treatment resulted in improved relapse-free survival and OS [447]. Nonetheless, the vitamin D analogue seocalcitol failed to demonstrate any objective anti-tumour activity in advanced pancreatic cancer [448]. A phase II study also reported no improvement with vitamin D supplementation in addition to chemotherapy in CRC [449]. Minnelide, a plant-derived compound, showed ability to deactivate CAFs and anti-tumour efficacy in preclinical models for pancreatic and liver cancers [450, 451]. Combining minnelide with chemotherapy led to a synergistic effect in pancreatic cancer models [452]. Clinical studies involving minnelide are ongoing, and no results have been reported. Angiotensin receptor blockers can also potentially reprogram CAFs into a quiescent state, and targeted delivery of angiotensin receptor blockers to tumours enhanced efficacy of immunotherapy in preclinical models [453]. While these therapies have shown promising results, clinical studies are so far limited.

## Conclusion and prospects

Significant progress has been made in the discovery and characterization of CAFs in the past. It is now widely acknowledged that CAFs play a pivotal role in tumour development and at least partially contribute to the failures of current anti-cancer therapies. Treatments targeting CAFs have been developed, and promising results have been observed in many preclinical studies. However, the translation of these CAF-targeted therapies into clinical interventions has proven challenging and has not been as successful as anticipated. A key obstacle is the absence of clinically relevant animal models to assess efficacy of CAF-targeted therapies. Unlike therapies directly targeting tumour cells, the effectiveness of CAF-targeted therapies largely depends on the microenvironment and the composition of tumour stroma in patients. Unfortunately, due to the complexity and heterogeneity of TME, these factors cannot be fully recapitulated in most preclinical models, leading to inconsistent outcomes of CAF-targeted therapies in animal models and patients. Cell line xenografts and allografts remain the most used models for examining CAF-targeted therapies in preclinical studies. To establish stromal abundant tumours in animal models, CAFs are often co-injected with tumour cells. However, the spatial distribution and phenotypes of these introduced CAFs may differ significantly from those observed in patients. Recent CAF classifications in cancer patients have identified diverse CAF subtypes with distinct functions, another complexity that many preclinical models fail to represent.

CAFs are a large and heterogeneous cell population within the intricate TME, playing complex roles in regulating tumour growth. Molecularly, CAFs interact with cancer cells and other stromal cells through secreted signalling molecules and receptors. They secrete a range of growth factors, chemokines, and cytokines that can directly affect receptors on cancer cells or other stromal cells, such as immune cells in the TME. Spatially, CAFs influence tumour growth by remodelling the ECM and forming physical barriers that impact tumour cell expansion and the infiltration of cells and treatments. These behaviours endow CAFs with multifaceted roles in cancers. The contribution of each characteristic to tumour growth may vary depending on the cancer type. For instance, in PDAC, the growth-inhibitory effect of the physical barrier formed by CAFs may outweigh the tumour-promoting effects of CAF-secreted factors. However, such physical barriers might also create niches that contribute to treatment resistance. Given the significant roles and high abundance of CAFs in tumours, targeting CAFs could be a potent strategy for treating cancers, especially when combined with other therapies. Nonetheless, treatment approaches should be carefully evaluated for different cancer types, and more innovative strategies are needed to eliminate their pro-tumour roles while preserving their tumour-restricting functions.

Current therapeutic approaches targeting CAFs primarily rely on utilisation of small inhibitors and antibodies. Nonetheless, these treatments exhibit a relatively modest inhibitory effect on CAFs, and resistance to such therapies could emerge over time. In response to these challenges, there has been a growing interest in using radioligand therapy or radiopharmaceutical therapy to deplete CAFs. These therapies have shown remarkable results in preclinical models, prompting the evaluation of treatments like ^177^Lu-FAPi in clinical settings. One notable advantage of radioligand therapy lies in its prolonged therapeutic effect, attributed to the long half-life of the delivered radioisotope. Moreover, the beta particle range of ^177^Lu enables these drugs to simultaneously inhibit growth of adjacent tumour cells [454]. This innovative approach presents a potential breakthrough in targeting CAFs with greater efficacy and sustained effects. Inspired by the development of ^177^Lu-FAPi, other radiopharmaceutical therapies targeting CAFs can be developed by radiolabelling existing CAF-targeted treatments with ^177^Lu or other suitable radioisotopes. These drugs may have superior CAF-ablating efficacy, as both the vehicle and carried radioisotopes contribute to the inhibition and depletion of CAFs.

Treatment strategies focusing on tumour-promoting factors derived from CAFs are appealing in scenarios where stromal barriers restrict cancer cell growth and movement. It is important to note, however, that targeting a single factor may only be successful in specific preclinical models and a limited subgroup of cancer patients where the specific factor plays a predominant role in promoting tumour growth. Given that CAFs can produce multiple tumour-promoting factors, these strategies are less likely to have a significant impact across broad cancer patients. The inhibitory effects of such therapies may be counterbalanced by increased expression of other tumour-promoting factors.

Most CAF-targeted therapies directly inhibit or regulate growth and behaviours of CAFs rather than tumours. Although these therapies can alter the TME and thereby affect tumour growth, their efficacy could be further enhanced when combined with other cancer treatments including chemotherapy, targeted therapy, and immunotherapy. However, the dosage, tolerability, and safety profiles of combination therapies should be carefully investigated. To reduce systemic toxicity caused by combination therapies and enhance tumour-specific targeting of the stromal cells, bispecific antibodies can be considered to concurrently target CAFs and cancer cells.

An expeditious approach for advancing development of CAF-targeted drugs is to repurpose existing non-cancer drugs already in clinical trials or approved by FDA. As activated fibroblasts in inflammatory conditions share similarities with CAFs, drugs with anti-fibrotic properties originally developed for conditions like idiopathic pulmonary fibrosis could be repurposed for inhibiting CAFs [455, 456]. This repurposing approach offers an accelerated pathway for developing CAF-targeted drugs, benefited from their established safety profiles and tolerability in other conditions.

While many clinical trials for CAF-targeted therapies primarily focus on patients with advanced and metastatic cancers, it will be worthwhile to explore the potential of these therapies in preventing cancer metastasis and relapse, as CAFs play essential roles in cancer cell dissemination and dormancy. Furthermore, assessing the feasibility of using CAF-targeted therapies as neoadjuvant treatments could open new avenues for future cancer treatment.

The accurate selection of patients is fundamental to ensuring the reliability and success of clinical trials for CAF-targeted therapies. Given the inherent heterogeneity of CAFs and individual variations, it is anticipated that these therapies will be effective in only a subset of cancer patients. Therefore, patients should be carefully selected based on reliable criteria, such as stroma-tumour ratio and target expression level.

## Data Availability

Not applicable.

## Abbreviations

TME: Tumour microenvironment
CAF: Cancer-associated fibroblast
TAM: Tumour-associated macrophage
Treg: Regulatory T
ECM: Extracellular matrix
scRNA-seq: Single-cell RNA sequencing
EMT: Epithelial-mesenchymal transition
KPC: LSL-Kras^G12D/+^;LSL-Trp53^R172H/+^;Pdx-1-Cre
SAA3: Serum Amyloid A3
LRCC15: Leucine rich repeat containing 15
FAP: Fibroblast activation protein
IFN: Interferon
TNF: Tumour necrosis factor
α-SMA: α-Smooth muscle actin
CSC: Cancer stem cell
HGF: Hepatocyte growth factor
SHH: Sonic hedgehog
PDGFR: Platelet-derived growth factor receptor
NF: Normal fibroblast
PDPN: Podoplanin
FSP-1: Fibroblast-specific protein 1
TN-C: Tenascin-C
POSTN: Periostin
PD-1: Programmed cell death protein 1
PD-L1: Programmed cell death ligand 1
Gal-1: Galectin-1
TKI: Tyrosine kinase inhibitor
CAV1: Caveolin 1
myCAF: Myofibroblastic CAF
iCAF: Inflammatory CAF
apCAF: Antigen-presenting CAF
HH: Hedgehog
HAS2: Hyaluronan synthase 2
FGF: Fibroblast growth factor
FAPI: FAP inhibitor
ICI: Immune checkpoint inhibitor.
CXCL: C-X-C motif chemokine ligand
CXCR: CXC receptor
ATRA: All-trans retinoic acid
OSCC: Oral squamous cell carcinoma
HCC: Hepatocellular carcinoma
PDAC: Pancreatic ductal adenocarcinoma
GI: Gastrointestinal
GBM: Glioblastoma
CRC: Colorectal cancer
NSCLC: Non-small cell lung cancer
SCLC: Small cell lung cancer
HNSCC: Head and neck squamous cell carcinoma
EAC: Oesophageal cancer
CRPC: Castration-resistant prostate cancer
SCC: Squamous cell carcinoma
ccRCC: Clear cell renal cell carcinoma
TNBC: Triple-negative breast cancer
MPM: Malignant pleural mesothelioma
BCC: Basal cell carcinoma
MM: Multiple myeloma
CCA: Cholangiocarcinoma
UCC: Urothelial carcinoma
GIST: Gastrointestinal stromal tumour
OS: Overall survival
DFS: Disease free survival
DCR: Disease control rate
ORR: Objective response rate
SD: Stable disease
PR: Partial response
PFS: Progression free survival
